# IMU-Based Automated Vehicle Slip Angle and Attitude Estimation Aided by Vehicle Dynamics †

**DOI:** 10.3390/s19081930

**Published:** 2019-04-24

**Authors:** Lu Xiong, Xin Xia, Yishi Lu, Wei Liu, Letian Gao, Shunhui Song, Yanqun Han, Zhuoping Yu

**Affiliations:** School of Automotive Studies, Tongji University, Shanghai 201804, China; luyishi_ysu@163.com (Y.L.); liuwei_ev@163.com (W.L.); letiangao_515@126.com (L.G.); songshunhui@foxmail.com (S.S.); yanqun_han@163.com (Y.H.); yuzhuoping@tongji.edu.cn (Z.Y.)

**Keywords:** sensor data fusion, slip angle estimation, attitude estimation, adaptive Kalman filter

## Abstract

The slip angle and attitude are vital for automated driving. In this paper, a systematic inertial measurement unit (IMU)-based vehicle slip angle and attitude estimation method aided by vehicle dynamics is proposed. This method can estimate the slip angle and attitude simultaneously and autonomously. With accurate attitude, the slip angle can be estimated precisely even though the vehicle dynamic model (VDM)-based velocity estimator diverges for a short time. First, the longitudinal velocity, pitch angle, lateral velocity, and roll angle were estimated by two estimators based on VDM considering the lever arm between the IMU and rotation center. When this information was in high fidelity, it was applied to aid the IMU-based slip angle and attitude estimators to eliminate the accumulated error correctly. Since there is a time delay in detecting the abnormal estimation results from VDM-based estimators during critical steering, a novel delay estimation and prediction structure was proposed to avoid the outlier feedback from vehicle dynamics estimators for the IMU-based slip angle and attitude estimators. Finally, the proposed estimation method was validated under large lateral excitation experimental tests including double lane change (DLC) and slalom maneuvers.

## 1. Introduction

Automated driving technology has attracted much attention recently [1]. Implementing high-level automated driving technology in on-road vehicles needs to address many cutting-edge issues. Among them, accurate sideslip angle and attitude are highly significant [2]. For example, image processing and feature recognition could be aided by the external pitch and roll angle of the vehicle body [3]. Also, sideslip angle and attitude are prerequisites for determining vehicle location [4]. From the perspective of vehicle dynamics control, slip angle, which has been researched for more than 20 years, is the basis for vehicle steering behavior control [5,6]. 

Unfortunately, commercial devices such as the RT3000 from OxTS [7] or the S-Motion from Kistler [8], which can measure vehicle slip angle and attitude, are too expensive to be used for commercial vehicles and are usually used only for experimental measurement purposes. The RT3000 is a GNSS/INS integration system which can estimate the vehicle velocity in navigation coordinates. Then the slip angle can be estimated by projecting the vehicle velocity in navigation coordinates onto the vehicle body coordinates. The S-Motion is an optical sensor to measure the slip angle. A more feasible way is to use an estimation technique by fusing information from different sensors of an intelligent vehicle. Usually, these sensors include lidars, radars, cameras, inertial measurement units (IMUs) and Global Navigation Satellite System (GNSS) receivers. Integrating the angular rate and acceleration sensor with attitude and velocity is one approach. However, angular rate and acceleration sensors are contaminated by unstable bias and long-term accumulated error when an automotive grade micro-electro-mechanical system (MEMS) IMU is used [9]. Thus, integrating a MEMS IMU to estimate slip angle and attitude should be done with the aid of other sensors such as a GNSS or cameras [10,11]. However, there are also some drawbacks when using IMUs with sensors like GNSS or cameras. The GNSS signal may be weak and easily blocked or suffer from multipath effects in city canyons. Cameras have high requirements for suitable light conditions. In dynamic situations such as emergency steering maneuvers, it is difficult to track the features all the time. In addition, delay and low sample rate issues emerge when low-cost GNSS and cameras are used. Much attention is required when implementing GNSS and cameras to estimate slip angle and attitude [12]. Besides GNSS and cameras, there are other standard sensors such as steering wheel angle and onboard angular speed sensors and wheel speed sensors. These sensors could be input to vehicle dynamics observers for slip angle estimation [6,13]. Using the estimated slip angle, the acceleration part due to translation movement could be removed and the residual part due to gravity could be used to calculate attitude [14]. However, in critical maneuvers, the performance of VDM–based observers would degrade due to model discrepancy. 

In Ref. [15], we proposed the elementary idea that, under normal driving conditions, the vehicle dynamic model (VDM)-based estimators for velocity and attitude could assist the IMU-based velocity and attitude estimators to remove the accumulated errors. In this paper, we improved the VDM-based estimators in lateral and longitudinal directions considering the lever arm between the IMU and the vehicle body rotation center. Besides, we further optimized the feedback mechanism for velocity and attitude and a systematic IMU-based vehicle slip angle and attitude estimation method aided by vehicle dynamics is proposed. The main contributions of this paper are summarized as follows:

- A novel and autonomous estimation method for slip angle and attitude without aids from external information such as GNSS or lane lines is proposed. The IMU-based slip angle and attitude estimator only needs assistance from VDM-based velocity and attitude estimators. Distinguished from many of the state of the art of slip angle estimation methods, which only consider horizontal motion, to further improve the estimation precision, especially in critical driving conditions, movement, including rotation and translation of the vehicle body in three dimensions, is considered. Simultaneous estimation of attitude and velocity keep the IMU-based estimator in a good state to prepare for open loop integration mode, when the vehicle enters critical driving conditions. An accurate attitude guarantees that the acceleration generated by gravity with changing attitude can be removed correctly. Then even when feedback from the VDM-based estimator is cut off, the estimation results of slip angle and attitude are still accurate for a short time.
- The proposed VDM-based estimator for attitude and velocity could eliminate the accumulated error of IMU-based slip angle and attitude estimation in normal driving conditions. Without accumulated error, the IMU-based slip angle and attitude estimation results have higher precision than the VDM-based estimators.
- A delayed estimator and predictor structure is proposed to deal with the time delay in detecting abnormal estimation results from VDM-based estimators. The delayed estimator and predictor structure avoids outlier feedback from the VDM-based estimators for IMU-based slip angle and attitude estimators.

The remainder of this paper is organized as follows: Section 2 introduces the state of the art. Section 3 explains the design procedure for the estimation method. Section 4 shows the experimental results. Finally, this paper is concluded in Section 5.

## 2. Related Work

Extensive work has been done to estimate slip angle and vehicle attitude. In this section, we provide a brief review of the state of the art. Attitude estimation methods can be divided into IMU-based and integration methods. Slip angle estimation methods can be divided into vehicle kinematic model (VKM)-based and VDM-based methods.

### 2.1. Attitude Estimation

The attitude can be represented by Euler angle, quaternion and direct cosine matrix [9]. In essence, they are similar ways of representing the attitude. The triaxial gyroscope was used to estimate attitude in direct cosine matrix and quaternion representation forms [16]. As discussed before, only a high-performance and high-cost gyroscope such as a ring-laser or fiber-optic gyroscope can generate accurate attitude estimation results in a relatively short time. Long-term integration without assistance from other sensors inevitably leads to huge accumulation error. Some studies have used the gravity part due to attitude from a triaxial accelerometer to calculate the pitch and roll angle to aid the gyroscope [9,17]. The main challenge in abstracting the attitude information from an accelerometer is variable acceleration due to translation movement. Threshold methods in Ref. [18] and fuzzy logic and adaptive methods in Ref. [19] were adopted to handle the translation movement problem. 

Other sensors could be used to give accessible measurement of attitude. A magnetometer can measure the direction of the sensor, but it is easily disturbed by surrounding magnetic materials [20]. Also, the pitch angle and roll angle are usually small and the signal-to-noise ratio is very low for magnetometers. Integrating GNSS for attitude estimation has been widely researched [10,21]; the main challenge is that the GNSS signal could be blocked by trees or high buildings, which limits its performance [22]. In recent years, cameras have been used to estimate vehicle attitude through computer vision [23]. Another limitation of GNSS and cameras is that their measurements are usually delayed and sampled at a low rate, which could cause extra measurement errors if the delay and low sample problems are not addressed [12].

### 2.2. Slip Angle Estimation

VKM–based methods use the kinematic relationship among sensors such as IMU, GNSS, or cameras to estimate slip angle. In Ref. [24], a direct integrator was used to estimate slip angle based on the basic relationship of sideslip angle and lateral acceleration. In Ref. [14], a six-degree-of-freedom (DOF) IMU was used to address the coupling problem between velocity and angular rate, and then a Luenberger-like observer was proposed to estimate the slip angle. In Ref. [25], an IMU combined with GNSS was chosen to estimate the sensor bias and slip angle. In Ref. [26], a camera was innovatively introduced to estimate the optical flow, and then the slip angle could be estimated. However, the accuracy of those kinds of observers depends on the output accuracy of the sensors. For example, sensors such as IMUs suffer from bias error, temperature drift, and random noise when measuring longitudinal and lateral acceleration and yaw rate. Besides, in critical situations, the accelerometer measurements contain the gravity component due to large roll angle and pitch angle [9]. More effort should be made to remove bias, noise, and the gravity part when integrating sensors. Otherwise huge estimation drift may arise after long-term integration. 

Besides those kinds of methods, the basic principle for VDM–based methods is to use somea measurable input signal, such as the steering wheel angle, driving torque, or braking torque exerted on the actuators, to drive a virtual vehicle dynamic model to generate lateral velocity. Then the measurable output of the actual vehicle, such as yaw rate and lateral acceleration, are used as feedback to correct the virtual slip angle, i.e., the estimated slip angle. Since different vehicle models, tire models, or estimation methods could be adopted to address different problems when estimating sideslip angle, many methods have arisen [6,13]. For example, in Ref. [24], a three-DOF vehicle model and magic formula tire model were used to describe the vehicle and tire dynamics. Then an unscented Kalman filter (UKF) was applied to estimate the sideslip angle. In Ref. [13], a variable structured extended Kalman filter (EKF) method was designed to estimate the sideslip angle based on a two-track vehicle model with three-DOF and magic formula tire model. Considering road friction, a high-gain observer was introduced to estimate the sideslip angle with small calculation load based on a single-track vehicle model [27]. However, the sideslip angle was hard to estimate due to the nonlinear and uncertain vehicle and tire dynamics [28]. The accuracy of those kinds of observers relies on the accuracy of the vehicle dynamic model. Model discrepancies will result in estimation errors. Some researchers have combined the kinematic model and dynamic model based methods to make full use of the merits of each one [6,11,29].

## 3. Methods

**Figure 1:**
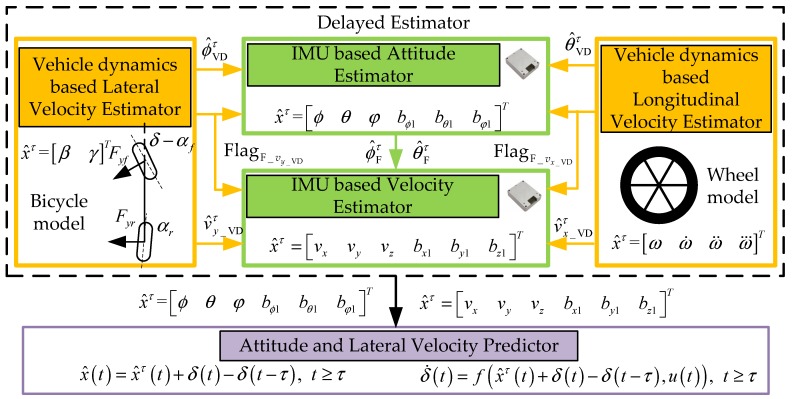
Overview of proposed slip angle and attitude estimation architecture. IMU, inertial measurement unit.

The holistic structure of the proposed estimation method in this paper is shown in Figure 1. The proposed estimation architecture is divided into two parts: the delayed estimator and the predictor. The delayed estimator contains two parts: the two IMU-based estimators, used to estimate velocity and attitude respectively, and the two VDM-based estimators, used to estimate longitudinal velocity and pitch angle and lateral velocity and roll angle respectively. In normal driving condition which can be determined by the ${\mathrm{Flag}}_{F\_v_{y\_\mathrm{VD}}}$ and ${\mathrm{Flag}}_{F\_v_{x\_\mathrm{VD}}}$, the feedback as measurement in Kalman filter from the VDM-based velocity and attitude estimators can remove the accumulated error of the IMU-based velocity and attitude estimators. In critical driving conditions such as fast steering or hard braking, the precision of the VDM-based estimation method is reduced significantly due to the model discrepancy. At this time, the IMU-based estimator should be insulated from the VDM-based estimator. However, the time delay needs to be detected to determine when to start the insulation, since the determination can only be made after the critical driving condition occurs. Therefore, those estimators are delayed to allow synchronization. In other words, those estimators are for estimating ${\hat{x}}^{\tau }\left(t\right)$ at t−τ moment. Then the predictor fuses the ${\hat{x}}^{\tau }\left(t\right)$ and presents input u(t) to predict the current $\hat{x}\left(t\right)$. 

### 3.1. Vehicle-Dynamics-Model-Based Velocity Estimator

This section shows the estimation methods of lateral velocity, roll angle, longitudinal velocity, and pitch angle by vehicle dynamics.

#### 3.1.1. Vehicle Kinematic Model

The kinematic model of the center of rotation is described by Equation (1):

(1) $$\left[\begin{array}{c} a_{x\_kine}\\ a_{y\_kine}\\ a_{z\_kine} \end{array}\right]=\left[\begin{array}{c} {\dot{v}}_{x}\\ {\dot{v}}_{y}\\ {\dot{v}}_{z} \end{array}\right]+\left[\begin{array}{c} \dot{\varphi }\\ \dot{\theta }\\ \dot{\psi } \end{array}\right]\times \left[\begin{array}{c} v_{x}\\ v_{y}\\ v_{z} \end{array}\right]$$

where $v_{x}$, $v_{y}$, and $v_{z}$ are longitudinal, lateral, and vertical velocity in vehicle body coordinates; $a_{x\_kine}$, $a_{y\_kine}$, and $a_{z\_kine}$ are kinematic acceleration of the vehicle body; and $\dot{\varphi }$, $\dot{\theta }$, and $\dot{\psi }$ are roll, pitch, and yaw angular velocity, respectively. However, due to the suspension, implementing the IMU at the rotation center is not possible, since there is a lever arm between the IMU and the rotation center. The lever arm will influence the measurement in the accelerometer when the vehicle body rotates. The lever arm should be estimated and then the convective acceleration should be removed from the acceleration. The convective acceleration is computed by Equation (2):

(2) $$\left[\begin{array}{c} a_{x\_\mathrm{compen}}\\ a_{y\_\mathrm{compen}}\\ a_{z\_\mathrm{compen}} \end{array}\right]=\left[\begin{array}{c} \ddot{\varphi }\\ \ddot{\theta }\\ \ddot{\psi } \end{array}\right]\times \left[\begin{array}{c} L_{\mathrm{offset}\_x}\\ L_{\mathrm{offset}\_y}\\ L_{\mathrm{offset}\_z} \end{array}\right]+\left[\begin{array}{c} \dot{\varphi }\\ \dot{\theta }\\ \dot{\psi } \end{array}\right]\times \left(\left[\begin{array}{c} \dot{\varphi }\\ \dot{\theta }\\ \dot{\psi } \end{array}\right]\times \left[\begin{array}{c} L_{\mathrm{offset}\_x}\\ L_{\mathrm{offset}\_y}\\ L_{\mathrm{offset}\_z} \end{array}\right]\right)$$

where $a_{x\_\mathrm{compen}}$,$a_{y\_\mathrm{compen}}$, and $a_{z\_\mathrm{compen}}$ are the compensated acceleration and $L_{\mathrm{offset}\_x}$, $L_{\mathrm{offset}\_y}$, and $L_{\mathrm{offset}\_z}$ are the lever arms in the *x*, *y*, and *z* directions, respectively; and $\ddot{\varphi }$, $\ddot{\theta }$, and $\ddot{\psi }$ are the roll, pitch, and yaw angular acceleration, respectively. 

Then we have the acceleration of the center of rotation of the body as Equation (3), where ${\left[\begin{array}{ccc} a_{x_{s}} & a_{y_{s}} & a_{z_{s}} \end{array}\right]}^{T}$ is the output of the accelerometer in *x*, *y*, and *z* directions, respectively:

(3) $$\left[\begin{array}{c} a_{x_{b}}\\ a_{y_{b}}\\ a_{z_{b}} \end{array}\right]=\left[\begin{array}{c} a_{x_{s}}\\ a_{y_{s}}\\ a_{z_{s}} \end{array}\right]-\left[\begin{array}{c} a_{x\_\mathrm{compen}}\\ a_{y\_\mathrm{compen}}\\ a_{z\_\mathrm{compen}} \end{array}\right]$$

Gravity will also contribute to the measurement in the accelerometer due to roll and pitch variation. The acceleration in body coordinate ${\left[\begin{array}{ccc} a_{x_{b}} & a_{y_{b}} & a_{z_{b}} \end{array}\right]}^{T}$ is given by Equation (4):

(4) $$\left[\begin{array}{c} a_{x_{b}}\\ a_{y_{b}}\\ a_{z_{b}} \end{array}\right]=\left[\begin{array}{c} a_{x\_kine}\\ a_{y\_kine}\\ a_{z\_kine} \end{array}\right]+\left[\begin{array}{c} -g\sin\theta \\ g\sin\varphi \cos\theta \\ g\cos\varphi \cos\theta \end{array}\right]$$

where *g* is gravity. The velocity ${\left[\begin{array}{ccc} {\hat{v}}_{x\_\mathrm{VD}} & {\hat{v}}_{y\_\mathrm{VD}} & {\hat{v}}_{z\_\mathrm{VD}} \end{array}\right]}^{T}$ and kinematic acceleration ${\left[\begin{array}{ccc} {\dot{\hat{v}}}_{x\_\mathrm{VD}} & {\dot{\hat{v}}}_{y\_\mathrm{VD}} & {\dot{\hat{v}}}_{z\_\mathrm{VD}} \end{array}\right]}^{T}$ could also be estimated from the vehicle dynamics (Equations (5), (9), (10), (25), and (26)). Based on this motivation, we can separate out the gravity component from the accelerometer to estimate the roll and pitch angles through Equation (6) to aid the IMU-based attitude estimator:

(5) $$\left[\begin{array}{c} a_{x_{kine}}\\ a_{y_{kine}}\\ a_{z_{kine}} \end{array}\right]=\left[\begin{array}{c} {\dot{\hat{v}}}_{x\_\mathrm{VD}}\\ {\dot{\hat{v}}}_{y\_\mathrm{VD}}\\ {\dot{\hat{v}}}_{z\_\mathrm{VD}} \end{array}\right]+\left[\begin{array}{c} {\dot{\varphi }}_{s}\\ {\dot{\theta }}_{s}\\ {\dot{\psi }}_{s} \end{array}\right]\times \left[\begin{array}{c} {\hat{v}}_{x\_\mathrm{VD}}\\ {\hat{v}}_{y\_\mathrm{VD}}\\ {\hat{v}}_{z\_\mathrm{VD}} \end{array}\right]$$

where ${\left[\begin{array}{ccc} {\dot{\varphi }}_{s} & {\dot{\theta }}_{s} & {\dot{\psi }}_{s} \end{array}\right]}^{T}$ is the angular velocity output from the gyroscope in the *x*, *y*, and *z* directions, respectively.

(6) $$\left[\begin{array}{c} a_{x_{bg}}\\ a_{y_{bg}}\\ a_{z_{bg}} \end{array}\right]=\left[\begin{array}{c} a_{x_{b}}\\ a_{y_{b}}\\ a_{z_{b}} \end{array}\right]-\left[\begin{array}{c} a_{x_{kine}}\\ a_{y_{kine}}\\ a_{z_{kine}} \end{array}\right]-\left[\begin{array}{c} a_{x\_\mathrm{compen}}\\ a_{y\_\mathrm{compen}}\\ a_{z\_\mathrm{compen}} \end{array}\right]=\left[\begin{array}{c} -g\sin\theta \\ g\sin\varphi \cos\theta \\ g\cos\varphi \cos\theta \end{array}\right]$$

where the subscript of *bg* means the acceleration due to gravity in body coordinate. With the gravity component in acceleration, the roll angle $\varphi_{\mathrm{VD}}$ and pitch angle $\theta_{\mathrm{VD}}$ can be computed as Equation (7):

(7) $$\left[\begin{array}{c} {\hat{\theta }}_{\mathrm{VD}}\\ {\hat{\varphi }}_{\mathrm{VD}} \end{array}\right]=\left[\begin{array}{c} -\arcsin(\frac{a_{x_{bg}}}{g})\\ \arcsin(\frac{a_{y_{bg}}}{g\cos\theta_{m\_delay}}) \end{array}\right]$$

#### 3.1.2. Longitudinal Velocity and Its Acceleration Estimation

There is much research about longitudinal velocity estimation based on vehicle dynamics and wheel dynamics. In this part, the wheel speed from the driven wheel is used to estimate the longitudinal velocity and longitudinal acceleration. In normal driving conditions, this wheel speed is accurate as the real longitudinal velocity, while in braking conditions, if the braking force is large, the wheel speed may diverge due to the tire slip. Thanks to the IMU-based attitude and velocity estimators, in strong braking conditions, the feedback from vehicle dynamics is cut off and the longitudinal velocity can be estimated with relatively high precision. 

• Longitudinal velocity estimation

**Figure 2:**
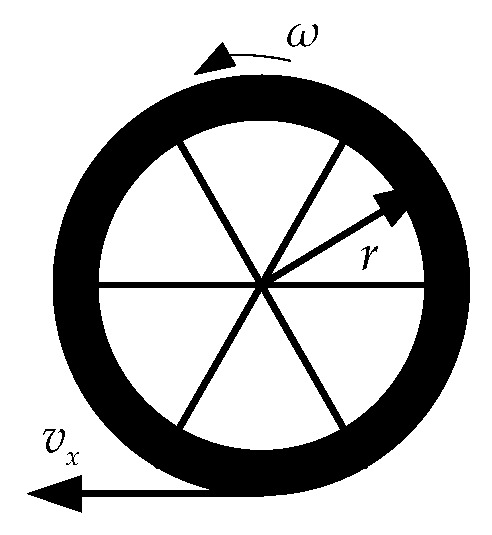
Wheel model.

The wheel model is shown in Figure 2. ω is rotation speed of the wheel and *r* is tire radius. The longitudinal velocity ${\hat{v}}_{x\_\mathrm{VD}}$ can be estimated from the wheel speed of the driven wheel as Equation (9):

(8) $$\left\{ \begin{array}{l} {\hat{v}}_{x\_rl}=\omega_{rl}\cdot r_{rl}+\frac{{\dot{\psi }}_{s}}{2}\cdot b_{r}\\ {\hat{v}}_{x\_rr}=\omega_{rr}\cdot r_{rr}-\frac{{\dot{\psi }}_{s}}{2}\cdot b_{r} \end{array}\right.$$

(9) $${\hat{v}}_{x\_\mathrm{VD}}=\frac{{\hat{v}}_{x\_rl}+{\hat{v}}_{x\_rr}}{2}$$

The subscripts *rl* and *rr* mean rear left and rear right, ω is the wheel speed, r is the tire radius, and $b_{r}$ is the rear wheel base.

• Kinematic longitudinal acceleration estimation

With the estimated longitudinal velocity, we can estimate the kinematic longitudinal acceleration to estimate the pitch angle. We assume that the longitudinal velocity can be described by a fourth-order Taylor series at t moment. Then, we have:

(10) $$\left\{ \begin{array}{ll} v_{x\_\mathrm{VD}}\left(t+△t\right) & =v_{x\_\mathrm{VD}}\left(t\right)+△t\cdot {\dot{v}}_{x\_\mathrm{VD}}\left(t\right)+\frac{1}{2!}△t^{2}\cdot {\ddot{v}}_{x\_\mathrm{VD}}\left(t\right)+\frac{1}{3!}△t^{3}\cdot {\dddot{v}}_{x\_\mathrm{VD}}\left(t\right)+O+w_{1}\\ {\dot{v}}_{x\_\mathrm{VD}}\left(t+△t\right) & ={\dot{v}}_{x\_\mathrm{VD}}\left(t\right)+△t\cdot {\ddot{v}}_{x\_\mathrm{VD}}\left(t\right)+\frac{1}{2!}△t^{2}\cdot {\dddot{v}}_{x\_\mathrm{VD}}\left(t\right)+O+w_{2}\\ {\ddot{v}}_{x\_\mathrm{VD}}\left(t+△t\right) & ={\ddot{v}}_{x\_\mathrm{VD}}\left(t\right)+△t\cdot {\dddot{v}}_{x\_\mathrm{VD}}\left(t\right)+O+w_{3}\\ {\dddot{v}}_{x\_\mathrm{VD}}\left(t+△t\right) & ={\dddot{v}}_{x\_\mathrm{VD}}\left(t\right)+w_{4} \end{array}\right.$$

where ${\dot{v}}_{x\_\mathrm{VD}}$, ${\ddot{v}}_{x\_\mathrm{VD}}$, and ${\dddot{v}}_{x\_\mathrm{VD}}$ are the first-order, second-order, and third-order derivatives of longitudinal speed; $w_{1}\sim w_{4}$ are Gaussian white noise; △t is the time step; and O is the high-order term, which is ignored. 

Remarks: Since the longitudinal acceleration is controlled by the driver, there would not be very high-order dynamics in the longitudinal velocity, and we make this assumption about the longitudinal velocity. 

Then, we have the discrete form of Equation (10). The system equation is given by Equation (11) and the measurement equation is given by Equation (12), where η means the measurement noise:

(11) $$\left[\begin{array}{c} v_{x\_\mathrm{VD}}\left(k+1\right)\\ {\dot{v}}_{x\_\mathrm{VD}}\left(k+1\right)\\ {\ddot{v}}_{x\_\mathrm{VD}}\left(k+1\right)\\ {\dddot{v}}_{x\_\mathrm{VD}}\left(k+1\right) \end{array}\right]=\left[\begin{array}{cccc} 1 & △t & \frac{1}{2!}△t^{2} & \frac{1}{3!}△t^{3}\\ 0 & 1 & △t & \frac{1}{2!}△t^{2}\\ 0 & 0 & 1 & △t\\ 0 & 0 & 0 & 1 \end{array}\right]\left[\begin{array}{c} v_{x\_\mathrm{VD}}\left(k\right)\\ {\dot{v}}_{x\_\mathrm{VD}}\left(k\right)\\ {\ddot{v}}_{x\_\mathrm{VD}}\left(k\right)\\ {\dddot{v}}_{x\_\mathrm{VD}}\left(k\right) \end{array}\right]+\left[\begin{array}{c} w_{1}\\ w_{2}\\ w_{3}\\ w_{4} \end{array}\right]$$

(12) $$v_{x\_\mathrm{VD}}=\left[\begin{array}{cccc} 1 & 0 & 0 & 0 \end{array}\right]\left[\begin{array}{c} v_{x\_\mathrm{VD}}\left(k\right)\\ {\dot{v}}_{x\_\mathrm{VD}}\left(k\right)\\ {\ddot{v}}_{x\_\mathrm{VD}}\left(k\right)\\ {\dddot{v}}_{x\_\mathrm{VD}}\left(k\right) \end{array}\right]+\eta$$

With different driving conditions such as passing deceleration strips or slipping, the noise in the wheel speed sensor changes a lot. The measurement noise covariance of wheel speed sensor output should be adapted with different driving conditions, which would enhance the dynamic performance of the filter. Then, the innovation adaptive estimation (IAE)-based Kalman filter mentioned in Section 3.1.3 is applied to estimate the longitudinal acceleration. 

• Feedback flag for IMU-based estimator

When a vehicle brakes hard, the tires will slip and the accuracy of the longitudinal velocity will drop fast. At this time, we need to detect this moment and insulate the feedback from the VDM-based longitudinal velocity estimator to the IMU-based longitudinal velocity estimator. Here, we design some feasible rules to set up the feedback flag for the IMU-based estimator. 

Define $a_{x\_\mathrm{dev}}$ as:

(13) $$a_{x\_\mathrm{dev}}=\left|{\dot{v}}_{x\_\mathrm{VD}}-\left(a_{x_{s}}-a_{x\_Compen}+g\sin\hat{\theta }\right)\right|$$

**Figure 3:**
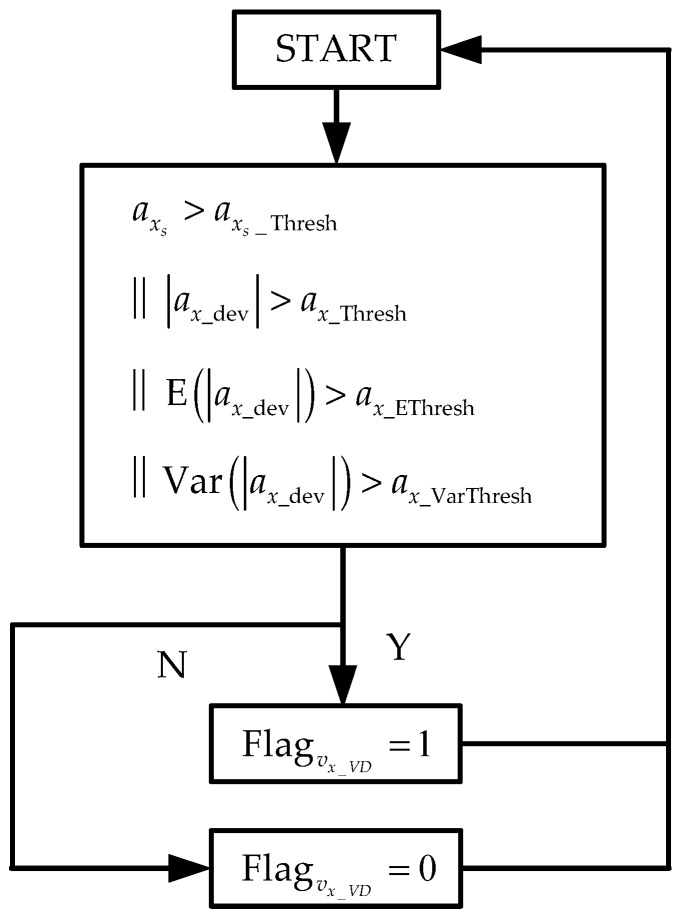
Feedback mechanism for longitudinal velocity and pitch angle estimator. $a_{x\_\mathrm{dev}}$ is the deviation between VDM-based and fused longitudinal acceleration; E means expectation; Var means variance for a short time; || means operation.

The mechanism of the feedback flag for the IMU-based longitudinal velocity and pitch angle estimator is shown in Figure 3. When the acceleration is smaller than a threshold value, ${\mathrm{Flag}}_{v_{x\_\mathrm{VD}}}$ is set up to detect the hard braking operation. When the expectation and variance of $a_{x\_\mathrm{dev}}$ are larger than the threshold value, ${\mathrm{Flag}}_{v_{x\_\mathrm{VD}}}$ is set up. $a_{x_{s}\_\mathrm{Thresh}}$, $a_{x\_\mathrm{Thresh}}$, $a_{x\_\mathrm{EThresh}}$, and $a_{x\_\mathrm{VarThresh}}$ are the threshold values that need to be tuned in the application. 

Essentially, the longitudinal velocity and pitch angle estimated from vehicle dynamics help to remove the accumulated error of the IMU-based estimator. Under normal driving conditions, the tire slip is very small, which means Equation (9) has high precision. This accurate longitudinal velocity and pitch angle is enough to remove the accumulated error. Thus, the threshold value in Figure 3 could be set strictly to detect abnormal values of the longitudinal velocity and pitch angle estimated from vehicle dynamics.

Since this mechanism is only effective when the tires have already slipped, if we use this flag to cut off the feedback to the IMU-based longitudinal velocity and attitude estimators, the IMU-based longitudinal velocity and attitude estimators have already been injected with polluted longitudinal velocity and pitch angle. In other words, this flag is too late to cut off the feedback, or there is a delay in the flag. Therefore, in order to synchronize, we delay the IMU-based estimator. As for the flag, we set up a new flag as Equation (14):

(14) $${\mathrm{Flag}}_{F\_v_{x\_\mathrm{VD}}}={\mathrm{Flag}}^{\tau }{}_{v_{x\_\mathrm{VD}}}∥{\mathrm{Flag}}_{v_{x\_\mathrm{VD}}}$$

where ${\mathrm{Flag}}^{\tau }{}_{v_{x\_\mathrm{VD}}}$ is delayed by τ from ${\mathrm{Flag}}_{v_{x\_\mathrm{VD}}}$.

This operation would guarantee the exact time to cut off the feedback, and we could use the longitudinal velocity and pitch angle from the vehicle dynamics estimator to assist the IMU-based attitude and longitudinal velocity estimator safely. In the end, the IMU-based estimator would output the delayed state.

#### 3.1.3. Estimation Algorithm 

**Figure 4:**
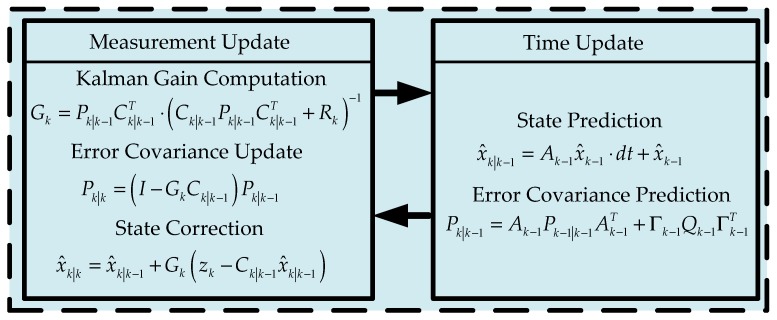
Kalman filter process.

In order to eliminate the random noise of the process model and measurement model, an IAE-based Kalman filter is proposed to estimate the velocity and attenuate the influence of the measurement outlier from the VDM-based estimator. The basic Kalman filter process is shown in Figure 4, where subscript k means the *k* moment, k|k−1 means prediction of *k* moment from *k*−1 moment, k|k means prediction after correction at time *k*, A is the system matrix, $\hat{x}$ is the state, P is the state error covariance, Q is the covariance of the system noise, Γ is the input matrix of the input noise, dt is the sample time, C is the measurement matrix, R is the covariance of measurement, G is the Kalman gain, and z is the measurement.

Since the noise of the measurement is usually time-varying, the noise covariance changes with different driving conditions. Therefore, the noise covariance should be adapted online to enhance the performance of the estimator. The innovation of the basic Kalman filter is given by Equation (15):

(15) $$d_{k}≐z_{k}-C_{k}{\hat{x}}_{k|k-1}$$

The expectation of the innovation at *k* moment is given by Equation (16):

(16) $$\begin{array}{ll} E\left(d_{k}\cdot d_{k}{}^{T}\right) & =E\left(\left(z_{k}-C_{k}{\hat{x}}_{k|k-1}\right){\left(z_{k}-C_{k}{\hat{x}}_{k|k-1}\right)}^{T}\right)\\ & =E\left(\left(C_{k}\left(x_{k|k}-{\hat{x}}_{k|k-1}\right)+\eta \right){\left(C_{k}\left(x_{k|k}-{\hat{x}}_{k|k-1}\right)+\eta \right)}^{T}\right)\\ & =E\left(C_{k}\left(x_{k|k}-{\hat{x}}_{k|k-1}\right){\left(x_{k|k}-{\hat{x}}_{k|k-1}\right)}^{T}C_{k}{}^{T}\right)+E\left(\eta \eta^{T}\right)\\ & =C_{k}P_{k|k-1}C_{k}{}^{T}+R_{k} \end{array}$$

$E\left(d_{k}\cdot d_{k}{}^{T}\right)$ can be computed through a short window [30]. Then the covariance of measurement noise is estimated by Equation (17):

(17) $$\begin{array}{ll} {\hat{R}}_{k} & =E\left(d_{k}\cdot d_{k}{}^{T}\right)-C_{k}P_{k|k-1}C_{k}{}^{T}\\ & =\frac{1}{n}\sum_{i=1}^{n}d_{k-i}\cdot d_{k-i}{}^{T}-C_{k}P_{k|k-1}C_{k}{}^{T} \end{array}$$

In order to reduce the calculation, we use the recursive form to compute ${\hat{R}}_{k}$, given by Equation (18):

(18) $$\begin{array}{ll} {\hat{R}}_{k} & =\sum_{i=1}^{k}\left(d_{i}\cdot d_{i}{}^{T}-C_{i}P_{i|i-1}C_{i}{}^{T}\right)\\ & =\frac{1}{k}\left[\left(\sum_{i=1}^{k-1}d_{i}\cdot d_{i}{}^{T}-C_{i}P_{i|i-1}C_{i}{}^{T}\right)+\left(d_{k}\cdot d_{k}{}^{T}-C_{k}P_{k|k-1}C_{k}{}^{T}\right)\right]\\ & =\frac{1}{k}\left[\left(k-1\right){\hat{R}}_{k-1}+\left(d_{k}\cdot d_{k}{}^{T}-C_{k}P_{k|k-1}C_{k}{}^{T}\right)\right]\\ & =\frac{k-1}{k}{\hat{R}}_{k-1}+\frac{1}{k}\left(d_{k}\cdot d_{k}{}^{T}-C_{k}P_{k|k-1}C_{k}{}^{T}\right) \end{array}$$

In order to improve the dynamic performance of the estimation for ${\hat{R}}_{k}$, we involve a fading factor to forget part of the historical measurement. The fading factor b is between 0 and 1; then, we have the fading coefficient $\alpha_{k}$:

(19) $$\alpha_{k}=\frac{\alpha_{k-1}}{\alpha_{k-1}+b}$$

The recursive form of the estimation for ${\hat{R}}_{k}$ is given by Equation (20):

(20) $${\hat{R}}_{k}=\left(1-\alpha_{k}\right){\hat{R}}_{k-1}+\alpha_{k}\left(d_{k}\cdot d_{k}{}^{T}-C_{k}P_{k|k-1}C_{k}{}^{T}\right)$$

In addition, since there is subtraction operation for $\left(d_{k}\cdot d_{k}{}^{T}-C_{k}P_{k|k-1}C_{k}{}^{T}\right)$, when $d_{k}$ and $P_{k|k-1}$ are mismatched, the sign of $\left(d_{k}\cdot d_{k}{}^{T}-C_{k}P_{k|k-1}C_{k}{}^{T}\right)$ may be negative, leading to the loss of positive certainty of ${\hat{R}}_{k}$, which would cause abnormity of the filter. Therefore, we add a limitation for every element of ${\hat{R}}_{k}$ for stability of the Kalman filter. For the *i-*th measurement, define $\chi_{k}^{i}$ as Equation (21):

(21) $$\chi_{k}^{i}={\left(d_{k}^{i}\right)}^{2}-C_{k}^{i}P_{k|k-1}^{i}C_{k}^{i}{}^{T}$$

Then ${\hat{R}}_{k}^{i}$ can be calculated as Equation (22):

(22) $${\hat{R}}_{k}^{i}=\left\{ \begin{array}{cc} \left(1-\alpha_{k}\right){\hat{R}}_{k-1}^{i}+\alpha_{k}{\hat{R}}_{\min}^{i} & \chi_{k}^{i}<{\hat{R}}_{\min}^{i}\\ {\hat{R}}_{\max}^{i} & \chi_{k}^{i}>{\hat{R}}_{\max}^{i}\\ \left(1-\alpha_{k}\right){\hat{R}}_{k-1}^{i}+\alpha_{k}\chi_{k}^{i} & {\hat{R}}_{\min}^{i}\leq \chi_{k}^{i}\leq {\hat{R}}_{\max}^{i} \end{array}\right.$$

In the following section, we will also use the IAE-based Kalman filter to estimate attitude and velocity by fusing vehicle dynamics and IMU information. 

#### 3.1.4. Lateral Velocity and Its Acceleration Estimation

As stated in the introduction, there is a lot of research about lateral velocity estimation based on vehicle dynamics [6,13]. In this section, a linear two-DOF single-track vehicle model is used to estimate the sideslip angle and lateral acceleration in normal driving conditions to remove the accumulated error of the IMU-based lateral velocity and attitude estimator.

• Lateral velocity estimation

**Figure 5:**
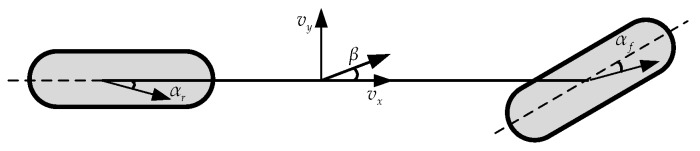
Single-track vehicle model.

In Figure 5, β is slip angle, $\alpha_{f}$ is tire slip angle of front axle and $\alpha_{r}$ is tire slip angle of rear axle. The dynamics of the linear 2DOF single-track vehicle model shown in Figure 5 is illustrated by Equation (23) [31]:

(23) $$\left[\begin{array}{c} \dot{\beta }\\ \ddot{\psi } \end{array}\right]=\underset{A}{\underset{︸}{\left[\begin{array}{cc} a_{11} & a_{12}\\ a_{21} & a_{22} \end{array}\right]}}\left[\begin{array}{c} \beta \\ \dot{\psi } \end{array}\right]+\underset{B}{\underset{︸}{\left[\begin{array}{c} b_{11}\\ b_{21} \end{array}\right]}}\delta_{f}$$

(24) $$\beta =\frac{v_{y}}{v_{x}}$$

where $C_{f}$ is front axle equivalent cornering stiffness, $C_{r}$ is rear axle equivalent cornering stiffness, m is vehicle mass, $l_{f}$ is distance from the front axle to the center of gravity (COG), $l_{r}$ is distance from the rear axle to the COG, $I_{z}$ is the vehicle yaw moment of inertia, $\delta_{f}$ is the steering angle of the front wheel, β is the sideslip angle, $A=\left[\begin{array}{cc} \frac{C_{f}+C_{r}}{mv_{x}} & \frac{C_{f}l_{f}-C_{r}l_{r}}{mv_{x}^{2}}-1\\ \frac{C_{f}l_{f}-C_{r}l_{r}}{I_{z}} & \frac{C_{f}l_{f}^{2}+C_{r}l_{r}^{2}}{I_{z}} \end{array}\right]$, and $B=\left[\begin{array}{c} -\frac{C_{f}}{mv_{x}}\\ -\frac{C_{f}l_{f}}{I_{z}} \end{array}\right]$.

Since we can obtain the yaw rate from the IMU, with that we can use the Kalman filter method shown in Figure 4 to estimate the sideslip angle. 

With the estimated sideslip angle, the lateral velocity can be estimated by Equation (25):

(25) $$v_{y\_\mathrm{VD}}=\beta \cdot v_{x}$$

• Kinematic lateral acceleration estimation

After updating the measurement of the sideslip angle estimation with the Kalman filter, we can also calculate the derivative of the sideslip angle based on the state equation:

(26) $${\dot{\hat{\beta }}}_{k|k}=\frac{C_{f}+C_{r}}{mv_{x}}{\hat{\beta }}_{k|k}+\left(\frac{C_{f}l_{f}-C_{r}l_{r}}{mv_{x}^{2}}-1\right)\dot{\psi }-\frac{C_{f}}{mv_{x}}\delta_{f}$$

In addition, the derivative of the lateral velocity is:

(27) $${\dot{\hat{v}}}_{y\_\mathrm{VD}}={\dot{\hat{\beta }}}_{k|k}\cdot v_{x}$$

• Feedback flag for IMU-based estimator

The design principle for the feedback flag for lateral velocity and roll angle is the same as that for longitudinal velocity. In the linear region of the tire, the linear tire model and 2DOF single-track vehicle model are well matched. With that, it is feasible to estimate the sideslip angle with high precision. As long as an accurate estimated sideslip angle is used, the accumulated error in the IMU-based lateral velocity and attitude estimator can be removed. 

We define $\gamma_{\mathrm{dev}}$, $v_{y\_\mathrm{dev}}$, and $a_{y\_\mathrm{dev}}$, which are the yaw rate deviation, lateral velocity deviation, and lateral kinematic acceleration deviation, respectively, as Equations (28)–(30). ${\hat{\gamma }}_{d}$ can be estimated from the linear 2DOF single-track vehicle model, as Equation (31).

(28) $$\gamma_{\mathrm{dev}}=\left|{\hat{\gamma }}_{d}-{\dot{\psi }}_{s}\right|$$

(29) $$v_{y\_\mathrm{dev}}=\left|{\hat{v}}_{y\_\mathrm{VD}}-{\hat{v}}_{y}\right|$$

(30) $$a_{y\_\mathrm{dev}}=\left|{\dot{\hat{v}}}_{y\_\mathrm{VD}}-\left(a_{y_{s}}-{\hat{v}}_{x}\dot{\psi }-g\sin\hat{\varphi }\cos\hat{\theta }\right)\right|$$

(31) $${\hat{\gamma }}_{d}=\frac{v_{x}/\left(l_{f}+l_{r}\right)}{1+\frac{m}{{\left(l_{f}+l_{r}\right)}^{2}}\left(\frac{l_{f}}{C_{r}}-\frac{l_{r}}{C_{f}}\right)v_{x}{}^{2}}\cdot \delta_{f}$$

**Figure 6:**
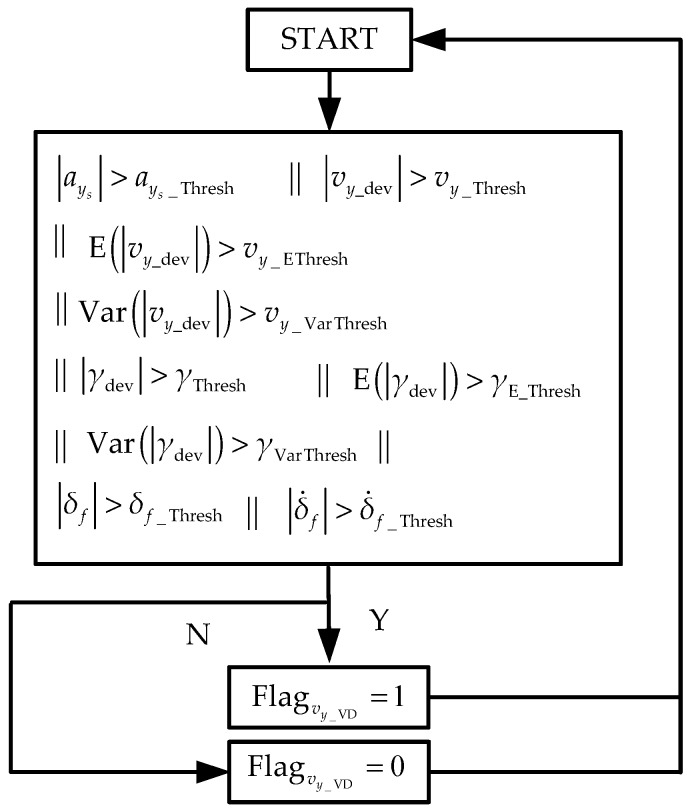
Feedback mechanism for lateral velocity and roll angle.

The mechanism of the feedback flag for the IMU-based lateral velocity and roll angle estimator is shown in Figure 6. When the lateral acceleration is larger than a threshold value, ${\mathrm{Flag}}_{v_{y\_\mathrm{VD}}}$ is set up to detect the critical steering operation. When the expectation and variance of $v_{y\_\mathrm{dev}}$, $\gamma_{\mathrm{dev}}$ are larger than the threshold value, ${\mathrm{Flag}}_{v_{y\_\mathrm{VD}}}$ is set up. In addition, the steering wheel angle and speed are also used to detect the critical steering operation. $a_{y_{s}\_\mathrm{Thresh}}$, $v_{y\_\mathrm{Thresh}}$, $v_{y\_\mathrm{EThresh}}$, $v_{y\_\mathrm{VarThresh}}$, $\gamma_{\mathrm{Thresh}}$, $\gamma_{E\_\mathrm{Thresh}}$, $\gamma_{\mathrm{Var}}{}_{\mathrm{Thresh}}$, $\delta_{f\_\mathrm{Thresh}}$, and ${\dot{\delta }}_{f\_\mathrm{Thresh}}$ are the threshold values that need to be tuned in application. The subscript of ’Thresh’ means threshold.

Like the longitudinal velocity estimation, only under normal driving conditions can the accumulated error in the IMU-based lateral velocity and roll angle estimator be removed. Thus, the threshold value in Figure 6 could be set strictly to detect abnormal values of the lateral velocity and roll angle estimated from vehicle dynamics.

Since this mechanism is only effective when the vehicle has already side-slipped, if we use this flag to cut off the feedback, the IMU-based lateral velocity and roll angle estimator have already been injected with polluted lateral velocity and roll angle. In other words, this flag is too late to cut off the feedback or there is a delay in the flag. Therefore, in order to synchronize, we delay the IMU-based estimator. As for the flag, we set up a new flag as Equation (32):

(32) $${\mathrm{Flag}}_{F\_v_{y\_\mathrm{VD}}}={\mathrm{Flag}}^{\tau }{}_{v_{y\_\mathrm{VD}}}∥{\mathrm{Flag}}_{v_{y\_\mathrm{VD}}}$$

where ${\mathrm{Flag}}^{\tau }{}_{v_{y\_\mathrm{VD}}}$ is delayed by τ from ${\mathrm{Flag}}_{v_{y\_\mathrm{VD}}}$.

This operation would guarantee the exact time to cut off the feedback, and we could use the lateral velocity and roll angle from the vehicle dynamics estimator to assist the IMU-based attitude and lateral velocity estimator safely. In the end, like the longitudinal velocity part, the IMU-based estimator would output the delayed state.

### 3.2. IMU-Based Attitude Estimation

In this section, based on the triaxle angular rate and the attitude estimated from the vehicle dynamics, we design the attitude estimator using the IAE- based Kalman filter.

#### 3.2.1. Gyroscope Sensor Model

We analyzed the gyroscope and acceleration sensor by the Allan variance method to determine the error composition in the IMU sensor [32]. The gyroscope or accelerometer measurement is composed of a real value, a constant bias term $b_{0}$, a random walk bias term $b_{1}$, and a wideband noise term w. A first-order Markov model can be used to show the random walk bias. τ is the time constant and $w_{b}$ is the wideband noise. Besides, due to the earth’s rotation, the gyroscope would also sense the angular speed ${\left[\begin{array}{ccc} {\dot{\varphi }}_{e} & {\dot{\theta }}_{e} & {\dot{\psi }}_{e} \end{array}\right]}^{T}$, and this part should be removed to estimate attitude. The gyroscope model is given by Equations (33) and (34):

(33) $$\left\{ \begin{array}{l} {\dot{\varphi }}_{s}={\dot{\varphi }}_{r}+b_{\varphi 0}+b_{\varphi 1}+{\dot{\varphi }}_{e}+w_{\varphi }\\ {\dot{\theta }}_{s}={\dot{\theta }}_{r}+b_{\theta 0}+b_{\theta 1}+{\dot{\theta }}_{e}+w_{\theta }\\ {\dot{\psi }}_{s}={\dot{\psi }}_{r}+b_{\psi 0}+b_{\psi 1}+{\dot{\psi }}_{e}+w_{\psi } \end{array}\right.$$

(34) $$\left\{ \begin{array}{l} {\dot{b}}_{\varphi 1}=-\frac{1}{\tau_{\varphi 1}}b_{\varphi 1}+w_{b_{\varphi 1}}\\ {\dot{b}}_{\theta 1}=-\frac{1}{\tau_{\theta 1}}b_{\theta 1}+w_{b_{\theta 1}}\\ {\dot{b}}_{\psi 1}=-\frac{1}{\tau_{\psi 1}}b_{\psi 1}+w_{b_{\psi 1}} \end{array}\right.$$

where the subscript *s* means the measurement of the sensor, the subscript *r* means the real measurement, and the superscript · means the derivative of the variable:

(35) $$\left[\begin{array}{c} {\dot{\varphi }}_{e}\\ {\dot{\theta }}_{e}\\ {\dot{\psi }}_{e} \end{array}\right]={\left[\begin{array}{ccc} \cos\psi \cos\theta & -\sin\psi \cos\varphi +\cos\psi \sin\theta \sin\varphi & \sin\psi \sin\varphi +\cos\psi \sin\theta \cos\varphi \\ \sin\psi \cos\theta & \cos\psi \cos\varphi +\sin\psi \sin\theta \sin\varphi & -\cos\psi \sin\varphi +\sin\psi \sin\theta \cos\varphi \\ -\sin\theta & \cos\theta \sin\varphi & \cos\theta \cos\varphi \end{array}\right]}^{T}\left[\begin{array}{c} 0\\ \omega_{ie}\cos L\\ \omega_{ie}\sin L \end{array}\right]$$

(36) $$\left[\begin{array}{c} {\dot{\varphi }}_{e}\\ {\dot{\theta }}_{e}\\ {\dot{\psi }}_{e} \end{array}\right]=\left[\begin{array}{ccc} \cos\psi & \sin\psi & 0\\ -\sin\psi & \cos\psi & 0\\ 0 & 0 & 1 \end{array}\right]\left[\begin{array}{c} 0\\ \omega_{ie}\cos L\\ \omega_{ie}\sin L \end{array}\right]$$

where $\omega_{ie}$ is the rotation speed of the Earth and *L* is the latitude of the vehicle. Equation (35) shows how to compute the angular speed in vehicle coordinates.

#### 3.2.2. Attitude Dynamics 

Since the simple form of Euler angle, in this paper, we chose the Euler angle as the representation of attitude. In Euler angle representation, we can involve the sensor bias in the state variable directly without further transformation compared with quaternion representation. The rotation sequence is *Z*-*Y*-*X*. Rotating about each axle, we have yaw, pitch, and roll angle, respectively. Then the dynamics of the Euler angle are given by Equation (37):

(37) $$\left[\begin{array}{c} \dot{\varphi }\\ \dot{\theta }\\ \dot{\psi } \end{array}\right]=\left[\begin{array}{ccc} 1 & \sin\varphi \tan\theta & \cos\varphi \tan\theta \\ 0 & \cos\varphi & -\sin\varphi \\ 0 & \sin\varphi /\cos\theta & \cos\varphi /\cos\theta \end{array}\right]\left[\begin{array}{c} {\dot{\varphi }}_{r}\\ {\dot{\theta }}_{r}\\ {\dot{\psi }}_{r} \end{array}\right]$$

#### 3.2.3. Attitude Estimator

For the attitude estimation, we applied the IAE-based Kalman filter. The state variable we used here contains ${\left[\begin{array}{cccccc} \varphi & \theta & \psi & b_{\varphi 1} & b_{\theta 1} & b_{\psi 1} \end{array}\right]}^{T}$ and the measurement variable is $\left[\begin{array}{ccc} \varphi_{m} & \theta_{m} & \psi_{m} \end{array}\right]$. The attitude dynamics can be described by Equation (38):

(38) $$\dot{x}\left(t\right)=f\left(x\left(t\right)\right)+\Gamma \left(x\left(t\right)\right)\xi \left(t\right)$$

where $x\left(t\right)={\left[\begin{array}{cccccc} \varphi & \theta & \psi & b_{\varphi 1} & b_{\theta 1} & b_{\psi 1} \end{array}\right]}^{T}$, $\xi \left(t\right)={\left[\begin{array}{cccccc} w_{\varphi } & w_{\theta } & w_{\psi } & w_{b_{\varphi 1}} & w_{b_{\theta 1}} & w_{b_{\psi 1}} \end{array}\right]}^{T}$, $f\left(x\left(t\right)\right)=\left[\begin{array}{c} {\dot{\varphi }}_{s}-(b_{\varphi 0}+b_{\varphi 1})+\sin\varphi \tan\theta \left({\dot{\theta }}_{s}-(b_{\theta 0}+b_{\theta 1}\right)+\cos\varphi \tan\theta \left({\dot{\psi }}_{s}-(b_{\psi 0}+b_{\psi 1})\right)\\ \cos\varphi \left({\dot{\theta }}_{s}-(b_{\theta 0}+b_{\theta 1})\right)-\sin\varphi \left({\dot{\psi }}_{s}-(b_{\psi 0}+b_{\psi 1})\right)\\ \sin\varphi /\cos\theta \left({\dot{\theta }}_{s}-(b_{\theta 0}+b_{\theta 1})\right)+\cos\varphi /\cos\theta \left({\dot{\psi }}_{s}-(b_{\psi 0}+b_{\psi 1})\right)\\ -\frac{1}{\tau_{\varphi 1}}b_{\varphi 1}\\ -\frac{1}{\tau_{\theta 1}}b_{\theta 1}\\ -\frac{1}{\tau_{\psi 1}}b_{\psi 1} \end{array}\right]$, and $\Gamma \left(x\left(t\right)\right)=\left[\begin{array}{cccccc} -1 & -\sin\varphi \tan\theta & -\cos\varphi \tan\theta & 0 & 0 & 0\\ 0 & -\cos\varphi & \sin\varphi & 0 & 0 & 0\\ 0 & -\sin\varphi /\cos\theta & -\cos\varphi /\cos\theta & 0 & 0 & 0\\ 0 & 0 & 0 & 1 & 0 & 0\\ 0 & 0 & 0 & 0 & 1 & 0\\ 0 & 0 & 0 & 0 & 0 & 1 \end{array}\right]$.

The model in Equation (38) is a nonlinear system, the EKF should be adopted [13]. The first step of the EKF is to compute the predicted state by Equation (39):

(39) $$x_{k|k-1}\approx f\left({\hat{x}}_{k}\right)△t+{\hat{x}}_{k}$$

Then in order to compute the state transition matrix, the system should be linearized. After the linearization given by Equation (40), the rest procedures of the EKF and KF are the same. The IAE-based Kalman filter is also implemented:

(40) $$\begin{array}{ll} {\dot{x}}_{k} & \approx f\left({\hat{x}}_{k}\right)+\frac{\partial f\left({\hat{x}}_{k}\right)}{\partial {\hat{x}}_{k}}\cdot \left(x_{k}-{\hat{x}}_{k}\right)\\ & =\frac{\partial f\left({\hat{x}}_{k}\right)}{\partial {\hat{x}}_{k}}\cdot x_{k}+\left[f\left({\hat{x}}_{k}\right)-\frac{\partial f\left({\hat{x}}_{k}\right)}{\partial {\hat{x}}_{k}}\cdot {\hat{x}}_{k}\right] \end{array}$$

The system matrix is given by Equation (41):

(41) $$A=\frac{\partial f\left(x\right)}{\partial x}=\left[\begin{array}{cc} A_{11} & A_{12}\\ A_{21} & A_{22} \end{array}\right]$$

where: 

$A_{11}=\left[\begin{array}{ccc} \cos\varphi \tan\theta \left({\dot{\theta }}_{s}-b_{\theta 1}\right)-\sin\varphi \tan\theta \left({\dot{\psi }}_{s}-b_{\psi 1}\right) & \frac{\sin\varphi }{\cos^{2}\theta }\left({\dot{\theta }}_{s}-b_{\theta 1}\right)+\frac{\cos\varphi }{\cos^{2}\theta }\left({\dot{\psi }}_{s}-b_{\psi 1}\right) & 0\\ -\sin\varphi \left({\dot{\theta }}_{s}-b_{\theta 1}\right)-\cos\varphi \left({\dot{\psi }}_{s}-b_{\psi 1}\right) & 0 & 0\\ \frac{\cos\varphi }{\cos\theta }\left({\dot{\theta }}_{s}-b_{\theta 1}\right)-\frac{\sin\varphi }{\cos\theta }\left({\dot{\psi }}_{s}-b_{\psi 1}\right) & \frac{\sin\varphi \sin\theta }{\cos^{2}\theta }\left({\dot{\theta }}_{s}-b_{\theta 1}\right)+\frac{\cos\varphi \sin\theta }{\cos^{2}\theta }\left({\dot{\psi }}_{s}-b_{\psi 1}\right) & 0 \end{array}\right]$, and $A_{12}=\left[\begin{array}{ccc} -1 & -\sin\varphi \tan\theta & -\cos\varphi \tan\theta \\ 0 & -\cos\varphi & \sin\varphi \\ 0 & -\sin\varphi /\cos\theta & -\cos\varphi /\cos\theta \end{array}\right]$, $A_{21}=\left[\begin{array}{ccc} 0 & 0 & 0\\ 0 & 0 & 0\\ 0 & 0 & 0 \end{array}\right]$, $A_{22}=\left[\begin{array}{ccc} -\frac{1}{\tau_{\varphi 1}} & 0 & 0\\ 0 & -\frac{1}{\tau_{\theta 1}} & 0\\ 0 & 0 & -\frac{1}{\tau_{{}_{\psi 1}}} \end{array}\right]$.

The measurement equation is

(42) $$\left[\begin{array}{c} {\hat{\varphi }}_{\mathrm{VD}}\\ {\hat{\theta }}_{\mathrm{VD}}\\ {\hat{\psi }}_{\mathrm{GNSS}} \end{array}\right]=\left[\begin{array}{cccccc} 1 & 0 & 0 & 0 & 0 & 0\\ 0 & 1 & 0 & 0 & 0 & 0\\ 0 & 0 & 1 & 0 & 0 & 0 \end{array}\right]\left[\begin{array}{c} \varphi \\ \theta \\ \psi \\ b_{\varphi 1}\\ b_{\theta 1}\\ b_{\psi 1} \end{array}\right]+\eta$$

where ${\hat{\psi }}_{\mathrm{GNSS}}$ is the heading angle from the GNSS receiver.

Then, with system matrix Equation (38) and measurement Equation (42), the IAE-based Kalman filter is used to estimate the attitude.

Remarks: As stated before, under critical driving conditions, the roll and pitch angles estimated from the vehicle dynamics lose fidelity and ${\mathrm{Flag}}_{F\_v_{x\_\mathrm{VD}}}$ and ${\mathrm{Flag}}_{F\_v_{y\_\mathrm{VD}}}$ are set. At this time, the feedback term should be cut off and the IMU-based attitude estimator turns to open loop integration mode.

### 3.3. IMU-Based Velocity Estimation

In this section, based on the triaxle accelerometer and the velocity estimated from the vehicle dynamics, we design the velocity estimator using the IAE-based Kalman filter.

#### 3.3.1. Accelerometer Sensor Model

Similar to the gyroscope sensor model, the accelerometer sensor model is given by Equations (43) and (44):

(43) $$\left\{ \begin{array}{l} a_{x_{s}}=a_{x_{r}}+b_{x0}+b_{x1}+w_{a_{x}}\\ a_{y_{s}}=a_{y_{r}}+b_{y0}+b_{y1}+w_{a_{y}}\\ a_{z_{s}}=a_{z_{r}}+b_{z0}+b_{z1}+w_{a_{z}} \end{array}\right.$$

(44) $$\left\{ \begin{array}{l} {\dot{b}}_{x1}=-\frac{1}{\tau_{x1}}b_{x1}+w_{b_{x1}}\\ {\dot{b}}_{y1}=-\frac{1}{\tau_{y1}}b_{y1}+w_{b_{y1}}\\ {\dot{b}}_{z1}=-\frac{1}{\tau_{z1}}b_{z1}+w_{b_{z1}} \end{array}\right.$$

#### 3.3.2. Velocity Dynamics

The dynamics of velocity in the vehicle body frame are given by Equation (45):

(45) $$\left[\begin{array}{c} {\dot{v}}_{x}\\ {\dot{v}}_{y}\\ {\dot{v}}_{z} \end{array}\right]=\left[\begin{array}{c} a_{x_{r}}\\ a_{y_{r}}\\ a_{z_{r}} \end{array}\right]-\left[\begin{array}{ccc} 0 & -{\dot{\psi }}_{r} & {\dot{\theta }}_{r}\\ {\dot{\psi }}_{r} & 0 & -{\dot{\varphi }}_{r}\\ -{\dot{\theta }}_{r} & {\dot{\varphi }}_{r} & 0 \end{array}\right]\left[\begin{array}{c} v_{x}\\ v_{y}\\ v_{z} \end{array}\right]-\left[\begin{array}{c} -g\sin\theta \\ g\sin\varphi \cos\theta \\ g\cos\varphi \cos\theta \end{array}\right]$$

#### 3.3.3. Velocity Estimator 

Since the vertical velocity is usually small under normal driving conditions, referring to the first-order Markov model, a damping term $-\frac{1}{\tau_{damp}}\cdot v_{z}$ is involved in the dynamics of vertical velocity in case of divergence. Therefore, the dynamics of velocity are changed to Equation (46):

(46) $$\begin{array}{ll} \left[\begin{array}{c} \begin{array}{c} {\dot{v}}_{x}\\ {\dot{v}}_{y}\\ {\dot{v}}_{z} \end{array}\\ {\dot{b}}_{x1}\\ {\dot{b}}_{y1}\\ {\dot{b}}_{z1} \end{array}\right] & =\left[\begin{array}{cccccc} 0 & {\dot{\psi }}_{s} & -{\dot{\theta }}_{s} & -1 & 0 & 0\\ -{\dot{\psi }}_{s} & 0 & {\dot{\varphi }}_{s} & 0 & -1 & 0\\ {\dot{\theta }}_{s} & -{\dot{\varphi }}_{s} & -\frac{1}{\tau_{damp}} & 0 & 0 & -1\\ 0 & 0 & 0 & -\frac{1}{\tau_{x1}} & 0 & 0\\ 0 & 0 & 0 & 0 & -\frac{1}{\tau_{y1}} & 0\\ 0 & 0 & 0 & 0 & 0 & -\frac{1}{\tau_{z1}} \end{array}\right]\left[\begin{array}{c} v_{x}\\ v_{y}\\ v_{z}\\ \begin{array}{c} b_{x1}\\ b_{y1}\\ b_{z1} \end{array} \end{array}\right]\\ & +\left[\begin{array}{cccccc} 1 & 0 & 0 & 0 & 0 & 0\\ 0 & 1 & 0 & 0 & 0 & 0\\ 0 & 0 & 1 & 0 & 0 & 0\\ 0 & 0 & 0 & 1 & 0 & 0\\ 0 & 0 & 0 & 0 & 1 & 0\\ 0 & 0 & 0 & 0 & 0 & 1 \end{array}\right]\left[\begin{array}{c} \left(a_{xs}-b_{x0}\right)+g\sin\hat{\theta }\\ \left(a_{ys}-b_{y0}\right)-g\sin\hat{\varphi }\cos\hat{\theta }\\ \left(a_{zs}-b_{z0}\right)-g\cos\hat{\varphi }\cos\hat{\theta }\\ 0\\ \begin{array}{c} 0\\ 0 \end{array} \end{array}\right]+\left[\begin{array}{cccccc} 1 & 0 & 0 & 0 & 0 & 0\\ 0 & 1 & 0 & 0 & 0 & 0\\ 0 & 0 & 1 & 0 & 0 & 0\\ 0 & 0 & 0 & 1 & 0 & 0\\ 0 & 0 & 0 & 0 & 1 & 0\\ 0 & 0 & 0 & 0 & 0 & 1 \end{array}\right]\left[\begin{array}{c} w_{a_{x}}\\ w_{a_{y}}\\ w_{a_{z}}\\ w_{b_{x1}}\\ w_{b_{y1}}\\ w_{b_{z1}} \end{array}\right] \end{array}$$

where the state variable is $x={\left[\begin{array}{cccccc} v_{x} & v_{y} & v_{z} & b_{x1} & b_{y1} & b_{z1} \end{array}\right]}^{T}$, input is ${\left[\begin{array}{cccccc} \left(a_{xs}-b_{x0}\right)+g\sin\hat{\theta } & \left(a_{ys}-b_{y0}\right)-g\sin\hat{\varphi }\cos\hat{\theta } & \left(a_{zs}-b_{z0}\right)-g\cos\hat{\varphi }\cos\hat{\theta } & 0 & 0 & 0 \end{array}\right]}^{T}$, and noise is ${\left[\begin{array}{cccccc} w_{a_{x}} & w_{a_{y}} & w_{a_{z}} & w_{b_{x1}} & w_{b_{y1}} & w_{b_{z1}} \end{array}\right]}^{T}$.

The measurement is given by Equation (47):

(47) $$\left[\begin{array}{c} v_{x\_\mathrm{VD}}\\ v_{y\_\mathrm{VD}} \end{array}\right]=\left[\begin{array}{cccccc} 1 & 0 & 0 & 0 & 0 & 0\\ 0 & 1 & 0 & 0 & 0 & 0 \end{array}\right]\left[\begin{array}{c} v_{x}\\ v_{y}\\ v_{z}\\ \begin{array}{c} b_{x1}\\ b_{y1}\\ b_{z1} \end{array} \end{array}\right]+\eta$$

Then, with system matrix Equation (46) and measurement Equation (47), the IAE-based Kalman filter is used to estimate the velocity.

As stated before, under critical driving conditions, the longitudinal and lateral velocity estimated from the vehicle dynamics lose fidelity and ${\mathrm{Flag}}_{F\_v_{x\_\mathrm{VD}}}$ and ${\mathrm{Flag}}_{F\_v_{y\_\mathrm{VD}}}$ are set. At this time, the feedback term should be cut off and the IMU-based velocity estimator turns to open loop integration mode. 

### 3.4. Attitude and Velocity Predictor

**Figure 7:**
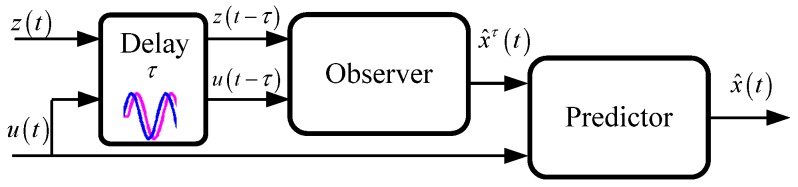
Delayed observer and predictor structure.

From Section 3.2, there is a time delay in ${\mathrm{Flag}}_{v_{x\_\mathrm{VD}}}$ and ${\mathrm{Flag}}_{v_{y\_\mathrm{VD}}}$, which are the indicators to insulate the IMU-based estimator from abnormal feedback from the VDM-based estimator. Therefore, we propose ${\mathrm{Flag}}_{F\_v_{x\_\mathrm{VD}}}$ and ${\mathrm{Flag}}_{F\_v_{y\_\mathrm{VD}}}$ to extend the time scale. When the IMU-based estimator is delayed to t−τ moment, ${\mathrm{Flag}}_{F\_v_{x\_\mathrm{VD}}}$ and ${\mathrm{Flag}}_{F\_v_{y\_\mathrm{VD}}}$ precede the delayed estimator to set up when the vehicle is under critical maneuvers, then a predictor is adopted to move the estimator at t−τ to *t* moment. This process is shown in Figure 7.

At *t* moment, the systems from Section 3.2, Section 3.3 and Section 3.4 for the estimator design can be abstracted by Equation (48), and the corresponding estimator is designed for estimation ${\hat{x}}^{\tau }\left(t\right)$ for t−τ moment.

(48) $$\begin{array}{l} \dot{x}\left(t-\tau \right)=f\left(x\left(t-\tau \right),u\left(t-\tau \right)\right)+w\left(t-\tau \right)\\ z\left(t-\tau \right)=h\left(x\left(t-\tau \right)\right)+\eta \left(t-\tau \right) \end{array}$$

${\hat{x}}^{\tau }\left(t\right)$ is the estimated result for t−τ moment at *t* moment. In real-time application, we should reserve a buffer to store the sensor data from t−τ to *t* for the predictor.

With ${\hat{x}}^{\tau }\left(t\right)$ (refer to Ref. [33]), a predictor is designed to predict $\hat{x}\left(t\right)$ based on the present *u*(*t*) and system model. The predictor is described by Equations (49) and (50):

(49) $$\dot{\delta }\left(t\right)=f\left({\hat{x}}^{\tau }\left(t\right)+\delta \left(t\right)-\delta \left(t-\tau \right),u\left(t\right)\right),\;\;t\geq \tau$$

(50) $$\hat{x}\left(t\right)={\hat{x}}^{\tau }\left(t\right)+\delta \left(t\right)-\delta \left(t-\tau \right),\;\;t\geq \tau$$

where the dynamics of δ(t) are the same as the corresponding system in Equation (48) and are driven by ${\hat{x}}^{\tau }\left(t\right)$ and u(t) with δ(t), $\hat{x}\left(t\right)$ predicted by Equation (50).

Within this delayed observer and predictor structure, the estimation error of the predictor is stable, and the proof of estimation error stability of the predictor is shown in Appendix A.

## 4. Results and Discussion

This section shows the experimental implementation and results.

### 4.1. Experimental Implementation

**Figure 8:**
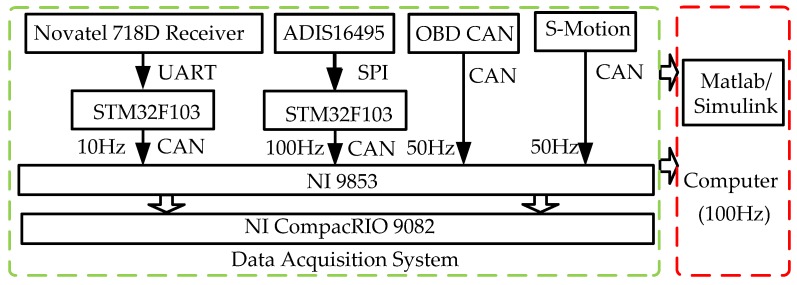
Hardware layout.

**Figure 9:**
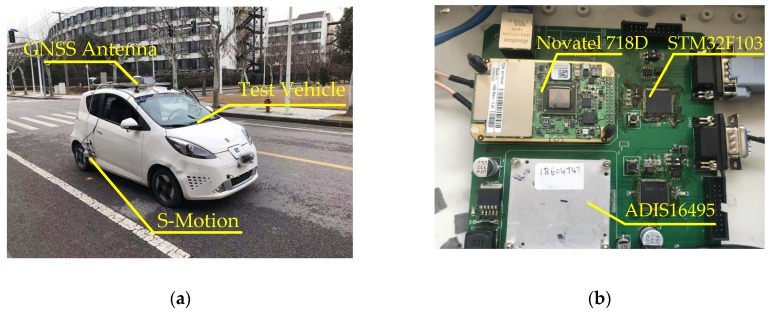
Hardware implementation: (**a**) test vehicle and part of equipment; (**b**) GNSS receiver and IMU.

Figure 8 and Figure 9 show the details of hardware implementation for this paper. The Novatel 718D receiver records the trajectory of the vehicle in 10 Hz and the ADIS 16495 provides the acceleration and angular speed and its increment in 100 Hz. The steering wheel angle, steering wheel angular speed, and wheel speed are acquired from the On-Board Diagnostics (OBD) port in the test vehicle in 50 Hz. The reference attitude, including roll angle, pitch angle, and velocity in longitudinal and lateral directions, are measured by the Kistler S-Motion in 50 Hz. All information is collected by the NI CompactRIO 9082 through a CAN bus and the data acquisition system is programmed by Labview 2013. MatLab/Simulink 2012a running on the computer is used to run the proposed method offline in 100 Hz. 

### 4.2. Expeimental Results

Double lane change (DLC) and slalom maneuvers under large lateral excitation were performed to validate the proposed estimation method. Large lateral excitation means lateral acceleration between 6 and 8 m/s^2^.

#### 4.2.1. DLC Maneuver 

**Figure 10:**
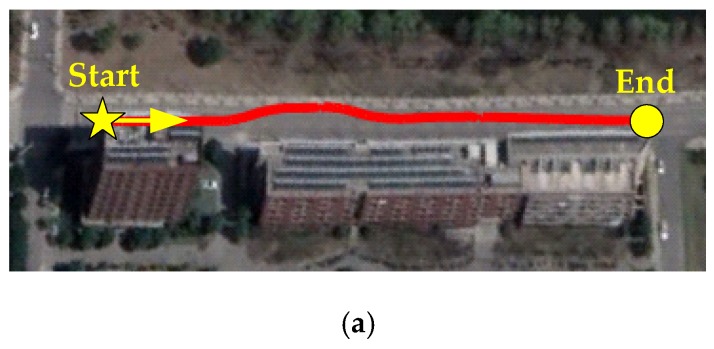

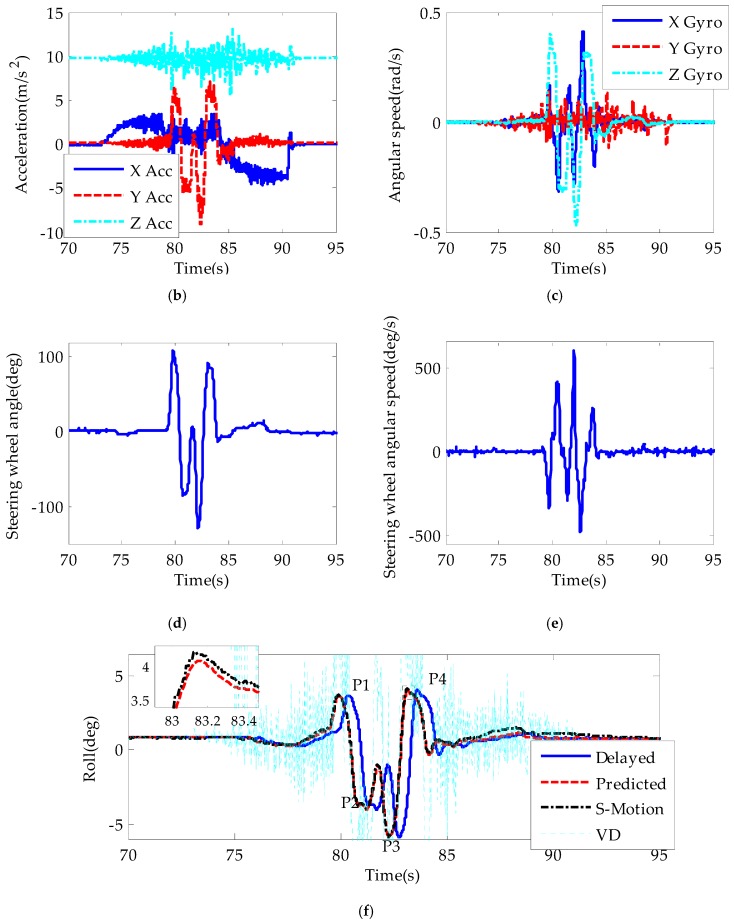

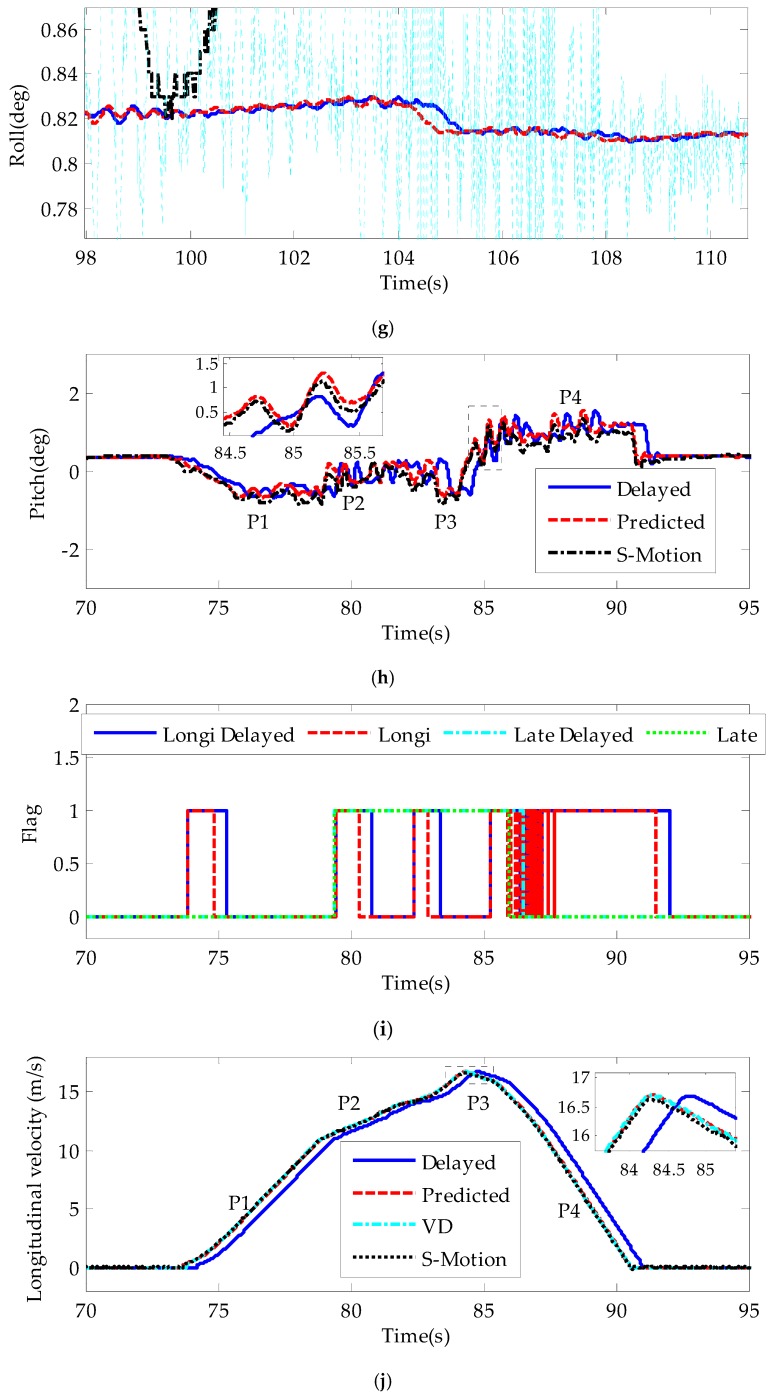

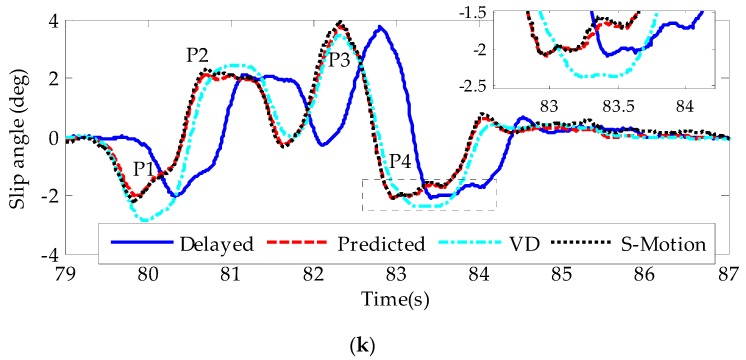
Test results in double lane change (DLC) maneuver: (**a**) trajectory; (**b**) acceleration; (**c**) angular speed; (**d**) steering wheel angle; (**e**) steering wheel angular speed; (**f**) roll angle; (**g**) partial enlarged detail of roll angle; (**h**) pitch angle; (**i**) flat; (**j**) longitudinal velocity; (**k**) slip angle.

Figure 10 shows the test results of the proposed method with the DLC maneuver. In the legend, late means lateral and longi means longitudinal; Delayed means the estimated result of ${\hat{x}}^{\tau }\left(t\right)$, Predicted means the estimation result of $\hat{x}\left(t\right)$ at the present moment, S-Motion is the measurement result from the Kistler S-Motion, and VD is the estimation result from the VDM-based estimator. Longi Delayed and Longi in the flag figure mean ${\mathrm{Flag}}_{F\_v_{x\_\mathrm{VD}}}$ and ${\mathrm{Flag}}_{v_{x\_\mathrm{VD}}}$, respectively. Late Delayed and Late in the flag figure mean ${\mathrm{Flag}}_{F\_v_{y\_\mathrm{VD}}}$ and ${\mathrm{Flag}}_{v_{y\_\mathrm{VD}}}$, respectively. ‘deg’ means degree. 

#### 4.2.2. Slalom Maneuver 

**Figure 11:**
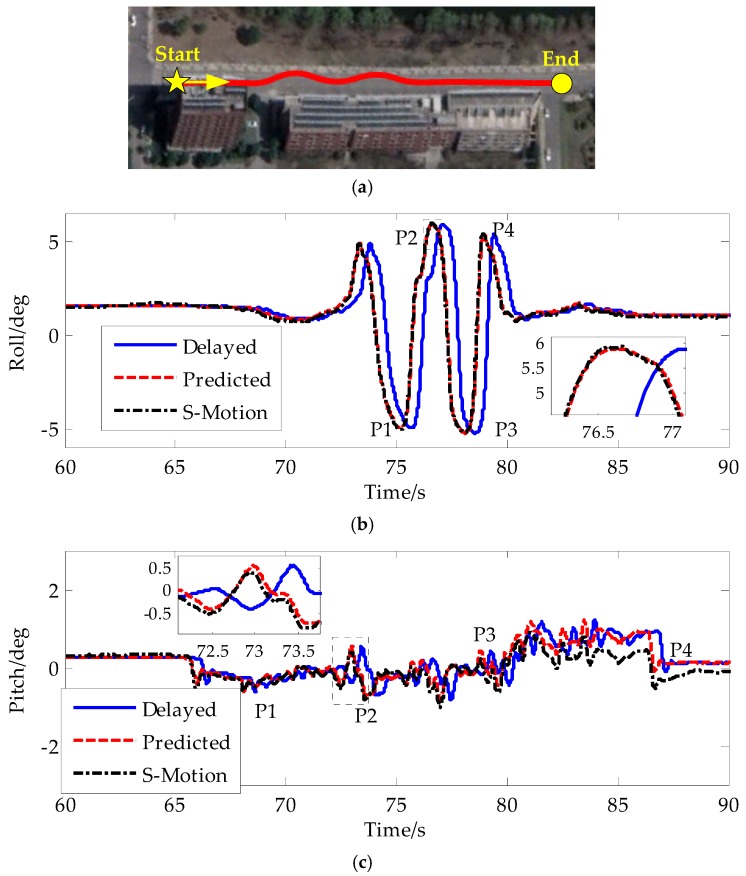

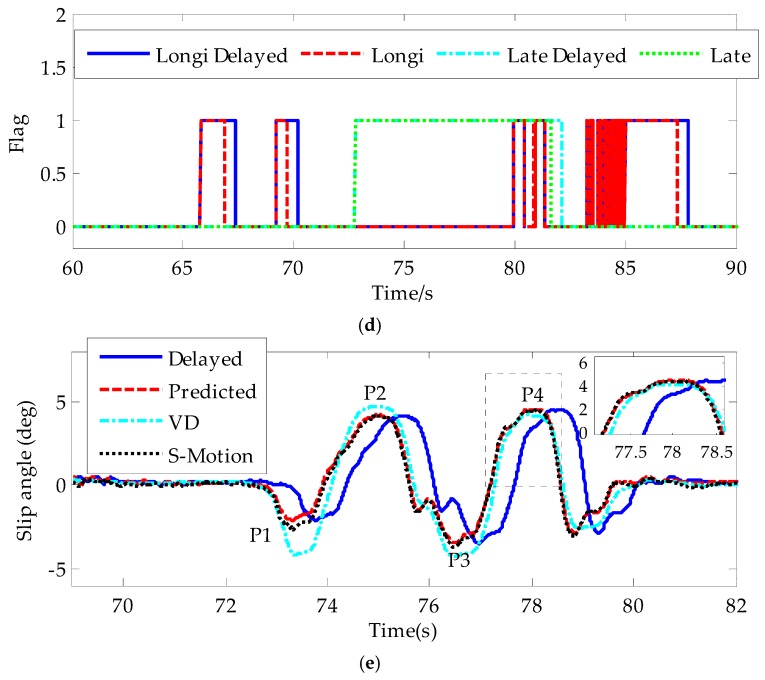
Test results in slalom maneuver: (**a**) trajectory; (**b**) roll angle; (**c**) pitch angle; (**d**) flag; (**e**) slip angle.

Figure 11 shows the test results of the proposed method with the slalom maneuver.

### 4.3. Discussion

Figure 10 and Figure 11 show the DLC and slalom maneuver test results. Figure 10a and Figure 11a show the vehicle trajectory. In those two maneuvers, the vehicle was driven violently with the steering wheel angular speed over 500°/s. The peak lateral acceleration reached 8 m/s^2^, which is nearly the road friction limit. Because the attitude from VDM-based estimators was very noise, which would affect the expression of the test results, the cyan line was only given in Figure 10f,g. In Figure 11, some of the important results were given compared with Figure 10. Table 1 and Table 2 show the absolute estimation errors in each maneuver. As for roll angle and slip angle, we select four peak points to compute the absolute estimation errors and averaged error. As for the pitch angle and longitudinal velocity, we randomly select four points to compute the absolute estimation errors and averaged error because under critical steering conditions, the dynamics in longitudinal direction is small. Table 3 and Table 4 show the root mean square errors in each maneuver. 

During the dramatic steering process, the vehicle body rotated fast with peak roll angle over 5°. Aided by the VDM-based estimator in normal driving conditions, the IMU-based attitude estimator could maintain a good state with roll angle estimation error smaller than 0.1° even without aid for a short time during the dramatic steering. The estimation error for pitch angle was below 0.5° in the total test process. The IAE-based Kalman filter for attitude estimation eliminated large noise, as shown by the green curves in Figure 10f,g. This is significant for the application from the control perspective. On the other hand, since the main error source of IMU-based estimator is the bias error, this accumulated error will not grow fast and in order to obtain the smooth estimation result, usually we set the noise covariance as a large initial value to reduce the weight of the measurement from VDM-based attitude estimators. Also the ${\hat{R}}_{\min}^{i}$ in Equation (22) is set as large value. From Figure 10f, we see that the cyan line which is the roll angle estimation result as the feedback for the IMU-based attitude estimation result was very noise. Thanks to the large noise covariance, the red line follows the cyan line smoothly in the partial enlarged detail of roll angle in Figure 10g. The convergence time is near 2 s from 0.82° to 0.8° from 104 s to 106 s. The averaged estimation error of roll angle was smaller than 0.1° and the RMS error of roll angle was smaller than 0.15°. The averaged estimation error of pitch angle was smaller than 0.2° and the RMS error of pitch angle was smaller than 0.2°. 

In normal driving conditions, the precise longitudinal and lateral velocity from the VDM-based estimator can be used as feedback to correct the IMU-based velocity estimator. This correction would remove the accumulated error in the IMU-based velocity estimator. From Figure 10j,k and Figure 11e, the velocity estimated by the proposed method can follow the velocity from the VDM-based estimator smoothly and without accumulated error. When the driver steered fast, the feedback to the lateral velocity was cut off if the flag in Figure 10i and Figure 11e was set. Then the slip angle shown in Figure 10k and Figure 11e was integrated in open loop integration mode. Thanks to the accurate attitude estimation result compensating the gravity component, the slip angle did not diverge over a short time. The maximum slip angle estimation error was less than 0.25° and the estimation precision reached 90%, which was much higher than that of the VDM-based estimator. This proved the idea in this paper that the VDM-based estimator would contribute bad information if we still injected it into the IMU-based estimator. Compared with lateral velocity, the precision of longitudinal velocity was higher because the tire slip ratio in the longitudinal direction was small, and it was over 95%. From Table 1 and Table 3, the averaged estimation error of longitudinal velocity was smaller than 0.1 m/s and the RMS error of longitudinal velocity was also smaller than 0.1 m/s from Table 2 and Table 4. The averaged estimation error of slip angle was smaller than 0.25° and the RMS error of slip angle was smaller than 0.1°, which was better than the slip angle from VDM-based estimator. 

The novel feedback strategy in Section 3.2.2 and Section 3.2.3 can generally detect critical driving conditions. Since the rules were established on the assumption that the vehicle had already entered into the critical situation, the detecting flag introduced a time delay to cut off the feedback. We proposed Equations (14) and (32) to extend the time domain of the critical driving condition as the blue and cyan curves shown in Figure 10i and Figure 11e, and in the meantime, we delayed all input and output to realize synchronization for a short time to make the flag setting precede the critical condition. Then we used the predictors to move the past state ${\hat{x}}^{\tau }\left(t\right)$ to the current time, as shown by the predicted curves in Figure 10f–k and Figure 11b–e, compared with the delayed curves. This moving process would not involve large estimation error in the current state, and this proof is theoretically made in Appendix A. 

## 5. Conclusions

In this paper, a novel and autonomous IMU-based vehicle slip angle and attitude estimation method aided by vehicle dynamics and GNSS is proposed. Three main conclusions can be drawn:

- Better performance has been gained by fusing VDM-based estimators and IMU-based estimators for slip angle and attitude than each of them. On the one hand, under normal driving conditions, assistance from VDM-based estimators can eliminate the accumulated error for the IMU–based slip angle and attitude estimation by the Kalman filter considering the lever arm between the IMU and rotation center. On the other hand, under critical driving conditions, without the accumulated error, the IMU-based slip angle and attitude estimation results have higher precision than the VDM-based estimator results.
- The simultaneous estimation of attitude and velocity keeps the IMU-based estimators in a good state to prepare for the open loop integration mode when the vehicle enters critical driving conditions. An accurate attitude guarantees that the acceleration generated by gravity with changing attitude can be removed correctly. Then, even when the feedback from the VDM-based estimators is cut off, the estimation results of slip angle and attitude are still accurate for a short time.
- The delayed estimator and predictor structure can avoid outlier feedback from VDM-based velocity and attitude estimators for IMU-based slip angle and attitude estimators with rejecting the time delay in detecting abnormal estimation results from VDM-based estimators. Also, the estimation error of the delayed estimator and predictor structure has been proved convergence theoretically.

In this paper, the aiding information for IMU-based estimators is obtained from estimators based on vehicle dynamics. In future work, our team will seek more accurate estimators based on vehicle dynamics to extend the aid to relatively critical driving conditions considering the nonlinear characteristics and uncertainty of the vehicle model. Also, when the IMU-based estimators enter the open loop integration mode under critical conditions, we will involve some external information from GNSS to correct the accumulated error. Last but not least, we will implement this method online on an embedded processor such as a DSP 28335.

## Figures and Tables

**Table 1:** Absolute estimation errors during critical steering in DLC maneuver. “Ave” means averaged estimation error. “P” means point.

	Proposed Method					Vehicle Dynamics				
	P1	P2	P3	P4	Ave	P1	P2	P3	P4	Ave
Roll angle (deg)	0.05	0.04	0.02	0.1	0.05	–	–	–	–	–
Pitch angle (deg)	0.05	0.26	0.2	0.25	0.19	–	–	–	–	–
Longi velocity (m/s)	0.11	0.02	0.06	0.15	0.09	0.05	0.08	0.01	0.02	0.04
Slip angle (deg)	0.01	0.21	0.15	0.02	0.10	0.91	0.65	0.42	0.45	0.61

**Table 2:** Absolute estimation errors during critical steering in slalom maneuver.

	Proposed Method					Vehicle Dynamics				
	P1	P2	P3	P4	Ave	P1	P2	P3	P4	Ave
Roll angle (deg)	0.09	0.01	0.02	0.07	0.05	–	–	–	–	–
Pitch angle (deg)	0.05	0.14	0.09	0.43	0.18	–	–	–	–	–
Longi velocity (m/s)	0.08	0.02	0.04	0.13	0.07	0.04	0.07	0.07	0.03	0.05
Slip angle (deg)	0.5	0.05	0.25	0.14	0.24	1.48	0.58	0.51	0.17	0.69

**Table 3:** Root mean square (RMS) of estimation errors in DLC maneuver.

	Proposed Method	Vehicle Dynamics
Roll angle (deg)	0.114	–
Pitch angle (deg)	0.168	–
Longi velocity (m/s)	0.054	0.032
Slip angle (deg)	0.069	0.176

**Table 4:** RMS of estimation errors in slalom maneuver.

	Proposed Method	Vehicle Dynamics
Roll angle (deg)	0.089	–
Pitch angle (deg)	0.181	–
Longi velocity (m/s)	0.05	0.03
Slip angle (deg)	0.100	0.291

## Appendix A

Assumption:

- The estimation error of ${\hat{x}}^{\tau }\left(t\right)$ is bounded.
- The system is Lipschitz with respect to *x*, which means there exists a positive constant L∈R such that $\left|f\left(x_{1},u\right)-f\left(x_{2},u\right)\right|\leq L\left|x_{1}-x_{2}\right|$ for any $x_{1}\in R$ and $x_{2}\in R$ and any u, where |⋅| means Euclidean norm on $R^{n}$.
- The delayed time τ is smaller than $\frac{1}{L}$ and there exists σ such that:
  (A1) $$L\frac{e^{\sigma \tau }-1}{\sigma }≺1$$

Proof:

Define the estimation error $e\left(t\right)≐\hat{x}\left(t\right)-x\left(t\right)$, and when t≥τ, we also have:

(A2) $$x\left(t\right)=x\left(t-\tau \right)+\int_{t-\tau }^{t}f\left(x\left(s\right),u\left(s\right)\right)ds$$

(A3) $$\begin{array}{ll} \hat{x}\left(t\right) & =\hat{x}\left(t-\tau \right)+\int_{t-\tau }^{t}f\left(\hat{x}\left(s\right),u\left(s\right)\right)ds\\ & ={\hat{x}}^{\tau }\left(t\right)+\int_{t-\tau }^{t}f\left(\hat{x}\left(s\right),u\left(s\right)\right)ds \end{array}$$

The initial state satisfies Equation (A4):

(A4) $$x\left(t\right)|\left[0,\tau \right]=\hat{x}\left(t\right)|\left[0,\tau \right]≐\hat{x}\left(\tau \right)+\delta \left(\tau \right)-\delta \left(0\right)$$

Subtract Equation (A2) from (A3) and then multiply the result by $e^{\sigma t}$, we have:

(A5) $$e^{\sigma t}\left|\hat{x}\left(t\right)-x\left(t\right)\right|\leq e^{\sigma t}\left(\left|{\hat{x}}^{\tau }\left(t\right)-x\left(t-\tau \right)\right|+\int_{t-\tau }^{t}\left|f\left(\hat{x}\left(s\right),u\left(s\right)\right)-f\left(x\left(s\right),u\left(s\right)\right)\right|ds\right)$$

With Inequality (A1), we have:

(A6) $$e^{\sigma t}\left|\hat{x}\left(t\right)-x\left(t\right)\right|\leq e^{\sigma t}\left(\left|{\hat{x}}^{\tau }\left(t\right)-x\left(t-\tau \right)\right|+L\int_{t-\tau }^{t}\left|\hat{x}\left(s\right)-x\left(s\right)\right|ds\right)$$

On the other hand, we get

(A7) $$\begin{array}{ll} \int_{t-\tau }^{t}\left|{\hat{x}}^{\tau }\left(s\right)-x\left(s\right)\right|ds= & \int_{t-\tau }^{t}e^{\sigma \left(s-s\right)}\left|{\hat{x}}^{\tau }\left(s\right)-x\left(s\right)\right|ds\\ & \leq \underset{t-\tau \leq s\leq t}{\sup}\left(e^{\sigma s}\left|\hat{x}\left(s\right)-x\left(s\right)\right|\right)\cdot \int_{t-\tau }^{t}e^{-\sigma s}ds \end{array}$$

However:

(A8) $$\int_{t-\tau }^{t}e^{-\sigma s}ds=e^{-\sigma t}\frac{e^{\sigma \tau }-1}{\sigma }\cdot$$

Therefore, we have:

(A9) $$\underset{t-\tau \leq s\leq t}{\sup}\left(e^{\sigma s}\left|{\hat{x}}^{\tau }\left(s\right)-x\left(s\right)\right|\right)\cdot \int_{t-\tau }^{t}e^{-\sigma s}ds=e^{-\sigma t}\frac{e^{\sigma \tau }-1}{\sigma }\cdot \underset{t-\tau \leq s\leq t}{\sup}\left(e^{\sigma s}\left|\hat{x}\left(s\right)-x\left(s\right)\right|\right)$$

With Inequality (A5) and Equation (A9) and $e^{-\sigma t}≺1$, we will get:

(A10) $$e^{\sigma t}\left|\hat{x}\left(t\right)-x\left(t\right)\right|\leq e^{\sigma t}\left|{\hat{x}}^{\tau }\left(t\right)-x\left(t-\tau \right)\right|+L\frac{e^{\sigma \tau }-1}{\sigma }\cdot \underset{t-\tau \leq s\leq t}{\sup}\left(e^{\sigma s}\left|\hat{x}\left(s\right)-x\left(s\right)\right|\right)$$

When t≥τ, taking the supremum over *t*, we will get:

(A11) $$\underset{\tau \leq s\leq t}{\sup}\left(e^{\sigma s}\left|\hat{x}\left(s\right)-x\left(s\right)\right|\right)\leq \underset{\tau \leq s\leq t}{\sup}\left(e^{\sigma s}\left|{\hat{x}}^{\tau }\left(s\right)-x\left(s-\tau \right)\right|\right)+L\frac{e^{\sigma \tau }-1}{\sigma }\cdot \underset{0\leq s\leq t}{\sup}\left(e^{\sigma s}\left|\hat{x}\left(s\right)-x\left(s\right)\right|\right)$$

Then two cases should be made to analyze the supremum.

(a) The supremum falls in [0,τ].

If Equation (A12) is satisfied, then Inequality (A11) becomes Inequality (A13):

(A12) $$\underset{0\leq s\leq t}{\sup}\left(e^{\sigma s}\left|\hat{x}\left(s\right)-x\left(s\right)\right|\right)=\underset{0\leq s\leq \tau }{\sup}\left(e^{\sigma s}\left|\hat{x}\left(s\right)-x\left(s\right)\right|\right)$$

(A13) $$\begin{array}{ll} \underset{\tau \leq s\leq t}{\sup}\left(e^{\sigma s}\left|\hat{x}\left(s\right)-x\left(s\right)\right|\right)\leq & \frac{\sigma }{\sigma -L\left(e^{\sigma \tau }-1\right)}\underset{\tau \leq s\leq t}{\sup}\left(e^{\sigma s}\left|{\hat{x}}^{\tau }\left(s\right)-x\left(s-\tau \right)\right|\right)\\ & +\underset{0\leq s\leq \tau }{\sup}\left(e^{\sigma s}\left|\hat{x}\left(s\right)-x\left(s\right)\right|\right) \end{array}$$

(b) The supremum falls in [τ,t].

If Equation (A14) is satisfied, then we also have Inequality (A13):

(A14) $$\underset{0\leq s\leq t}{\sup}\left(e^{\sigma s}\left|\hat{x}\left(s\right)-x\left(s\right)\right|\right)=\underset{\tau \leq s\leq t}{\sup}\left(e^{\sigma s}\left|\hat{x}\left(s\right)-x\left(s\right)\right|\right)$$

According to Inequality (A13) and combined with $e^{\sigma t}\left|\hat{x}\left(t\right)-x\left(t\right)\right|\leq \underset{\tau \leq s\leq t}{\sup}\left(e^{\sigma s}\left|\hat{x}\left(s\right)-x\left(s\right)\right|\right)$, multiplying $e^{-\sigma t}$ by Equation (A12), we have:

(A15) $$\begin{array}{ll} \left|\hat{x}\left(t\right)-x\left(t\right)\right| & \leq e^{-\sigma t}\underset{\tau \leq s\leq t}{\sup}\left(e^{\sigma s}\left|\hat{x}\left(s\right)-x\left(s\right)\right|\right)\\ & \leq \frac{\sigma }{\sigma -L\left(e^{\sigma \tau }-1\right)}\underset{\tau \leq s\leq t}{\sup}\left(e^{\sigma \left(s-t\right)}\left|{\hat{x}}^{\tau }\left(s\right)-x\left(s-\tau \right)\right|\right)+\underset{0\leq s\leq \tau }{e^{-\sigma t}\sup}\left(e^{\sigma s}\left|\hat{x}\left(s\right)-x\left(s\right)\right|\right) \end{array}$$

Therefore, we get that the estimation error converges to an upper bound given by Inequality (A16):

(A16) $$\left|e\left(t\right)\right|\leq \frac{\sigma }{\sigma -L\left(e^{\sigma \tau }-1\right)}\underset{\tau \leq s\leq t}{\sup}\left(e^{\sigma \left(s-t\right)}e\left(s-\tau \right)\right)+\underset{0\leq s\leq \tau }{e^{-\sigma t}\sup}\left(e^{\sigma s}e\left(s\right)\right)$$

From Inequality (A16), the first part on the right side is related to τ and the second part is related to the initial estimation error. There is an attenuation term $e^{-\sigma t}$ in the second part, therefore the second part would converge to zero over time.

In addition, with Equation (A16) we can draw three conclusions: if the estimation error of ${\hat{x}}^{\tau }\left(t\right)$ is bounded, then e(t) is bounded; if the estimation error of ${\hat{x}}^{\tau }\left(t\right)$ is asymptotically convergent, then e(t) is asymptotically convergent; if the estimation error of ${\hat{x}}^{\tau }\left(t\right)$ is exponentially convergent, then e(t) is exponentially convergent.

(i) e(t) is bounded.

If the estimation error of ${\hat{x}}^{\tau }\left(t\right)$ is smaller than $\delta_{0}\geq 0$ and t≥τ, from Inequality (A16), it is easy to derive Inequality (A17) with respect to any t≥τ:

(A17) $$\left|e\left(t\right)\right|\leq \frac{\sigma }{\sigma -L\left(e^{\sigma \tau }-1\right)}\delta_{0}+\delta_{1}$$

(ii) e(t) is asymptotically convergent.

If the estimation error of ${\hat{x}}^{\tau }\left(t\right)$ satisfies $\underset{t\to \infty }{\lim}e\left(t-\tau \right)=0$, then for any $\varsigma_{r}≻0$, there exists $T_{r}$ such that, for any $t\geq T_{r}$, we have $e\left(t-\tau \right)\leq \varsigma_{r}$. Thus for any $t\geq T_{r}$, from Inequality (A16), we have Inequality (A18):

(A18) $$\begin{array}{ll} \left|e\left(t\right)\right| & \leq \frac{\sigma }{\sigma -L\left(e^{\sigma \tau }-1\right)}\varsigma_{r}\underset{\tau \leq s\leq t}{\sup}\left(e^{\sigma \left(s-t\right)}\right)+e^{-\sigma t}\underset{0\leq s\leq \tau }{\sup}\left(e^{\sigma s}e\left(s\right)\right)\\ & ≺\frac{\sigma }{\sigma -L\left(e^{\sigma \tau }-1\right)}\varsigma_{r}+e^{-\sigma t}\underset{0\leq s\leq \tau }{\sup}\left(e^{\sigma s}e\left(s\right)\right) \end{array}$$

With specified ς≻0, we can always find $\varsigma_{r}$ small enough and $T^{*}$ large enough such that

(A19) $$\frac{\sigma }{\sigma -L\left(e^{\sigma \tau }-1\right)}\varsigma_{r}+\underset{\;\;\;\;\;\;\;\;\;\;\;\;0\leq s\leq \tau }{e^{-\sigma T^{*}}\sup}\left(e^{\sigma s}e\left(s\right)\right)$$

Therefore, for any $t\geq \max\left(T^{*},T_{r}\right)$, we have |e(t)|≺ς, which means Equation (A20) holds:

(A20) $$\underset{t\to \infty }{\lim}e\left(t\right)=0$$

(iii) e(t) is exponentially convergent.

If the estimation error of ${\hat{x}}^{\tau }\left(t\right)$ is exponentially convergent, there exist positive κ and χ such that:

(A21) $$e\left(t-\tau \right)\leq \kappa \left|{\hat{x}}^{\tau }\left(\tau \right)-x\left(0\right)\right|e^{-\chi t}$$

Then the first part on the right side of Inequality (A16) satisfies Inequality (A22):

(A22) $$\begin{array}{ll} \underset{\tau \leq s\leq t}{\sup}\left(e^{\sigma \left(s-t\right)}e\left(s-\tau \right)\right) & \leq \kappa \left|{\hat{x}}^{\tau }\left(\tau \right)-x\left(0\right)\right|e^{-\left(\chi +\sigma \right)t}\cdot \underset{\tau \leq s\leq t}{\sup}\left(e^{\sigma s}\right)\\ & \leq \kappa \left|{\hat{x}}^{\tau }\left(\tau \right)-x\left(0\right)\right|e^{-\chi t} \end{array}$$

Substituting Inequality (A21) into Inequality (A16), we have:

(A23) $$\begin{array}{ll} \left|e\left(t\right)\right| & \leq \frac{\sigma }{\sigma -L\left(e^{\sigma \tau }-1\right)}\kappa \left|{\hat{x}}^{\tau }\left(\tau \right)-x\left(0\right)\right|e^{-\chi t}+e^{-\sigma t}\underset{0\leq s\leq \tau }{\sup}\left(e^{\sigma s}e\left(s\right)\right)\\ & \leq \bar{\kappa }e^{-\bar{\chi }t} \end{array}$$

where $\bar{\chi }=\min\left(\sigma ,\chi \right)$ and $\bar{\kappa }=\frac{\sigma }{\sigma -L\left(e^{\sigma \tau }-1\right)}\kappa \left|{\hat{x}}^{\tau }\left(\tau \right)-x\left(0\right)\right|e^{\left(\bar{\chi }-\chi \right)\tau }+\underset{0\leq s\leq \tau }{\sup}\left(e^{\sigma s}e\left(s\right)\right)e^{\left(\left(\bar{\chi }-\sigma \right)\tau \right)}$.

Thus, if there is a convergent estimation error in ${\hat{x}}^{\tau }\left(t\right)$, the estimation error of $\hat{x}\left(t\right)$ is also convergent.
